# Angiogenesis and inflammation in the retinopathy risk of insulin and semaglutide – a review

**DOI:** 10.1186/s40942-026-00811-8

**Published:** 2026-03-18

**Authors:** Ming Lu

**Affiliations:** Christus Trinity Clinic Eye Center, 1327 Troup Highway, Tyler, TX 75701 USA

**Keywords:** Semaglutide, Insulin, Diabetic retinopathy, Glucagon-like peptide-1 receptor agonist, Angiogenesis

## Abstract

**Purpose:**

To critically evaluate evidence supporting the angiogenesis-inflammation hypothesis in diabetic retinopathy (DR); to examine the roles of angiogenic growth factors and inflammatory cytokines in the biphasic effects of insulin on DR - early worsening followed by long-term risk reduction; and to explore broader implications of this hypothesis, including potential biphasic effects of semaglutide due to its insulinotropic action and strategies to mitigate the transient DR exacerbation associated with insulin and insulin secretagogues.

**Methods:**

Literature review.

**Results:**

Hyperglycemia and the accumulation of advanced glycation end products upregulate angiogenic growth factors and inflammatory cytokines, notably vascular endothelial growth factor (VEGF) and angiopoietin-2. These mediators compromise the retinal neurovascular unit and disrupt the blood-retinal barrier, while promoting endothelial adhesion molecule expression. The resulting leukostasis triggers hypoxia, leukocyte activation, and self-perpetuating ischemic-inflammatory loops. Insulin-like growth factor-1 further enhances retinal VEGF and angiopoietin-2 expression, potentially driving DR to the proliferative stage. Semaglutide, a glucagon-like peptide-1 (GLP-1) receptor agonist, has paradoxically been linked to DR deterioration like insulin therapy. In contrast, insulin-independent antidiabetic agents such as empagliflozin and metformin are not associated with DR worsening and may even slow its progression. GLP-1 augments glucose-induced insulin secretion, whereas insulin itself upregulates retinal VEGF and angiopoietin-2 and exerts pro-inflammatory effects in insulin-resistant states. Despite early worsening, long-term insulin therapy reduces DR risk through sustained glycemic control, with benefits outweighing initial harm. Semaglutide addresses a broader spectrum of systemic risk factors for DR than insulin, including hypertension, dyslipidemia, chronic kidney disease, and obesity. In addition, experimental evidence suggests that semaglutide may provide direct neurovascular protection through activation of the retinal GLP-1 receptor.

**Conclusions:**

The early worsening of DR associated with insulin therapy may be more accurately attributed to insulin’s pro-angiogenic effects rather than solely to rapid glycemic correction. As an insulin secretagogue, semaglutide may similarly cause early DR progression. However, long-term semaglutide therapy is anticipated to reduce DR risk by improving glycemic control, addressing a broader range of systemic risk factors compared with insulin, and offering direct neurovascular protection in the retina. Combination therapy with insulin-independent glucose-lowering agents may help mitigate the transient DR deterioration linked to insulin and insulin secretagogues.

**Supplementary Information:**

The online version contains supplementary material available at 10.1186/s40942-026-00811-8.

## Introduction

**Fig. 1:**
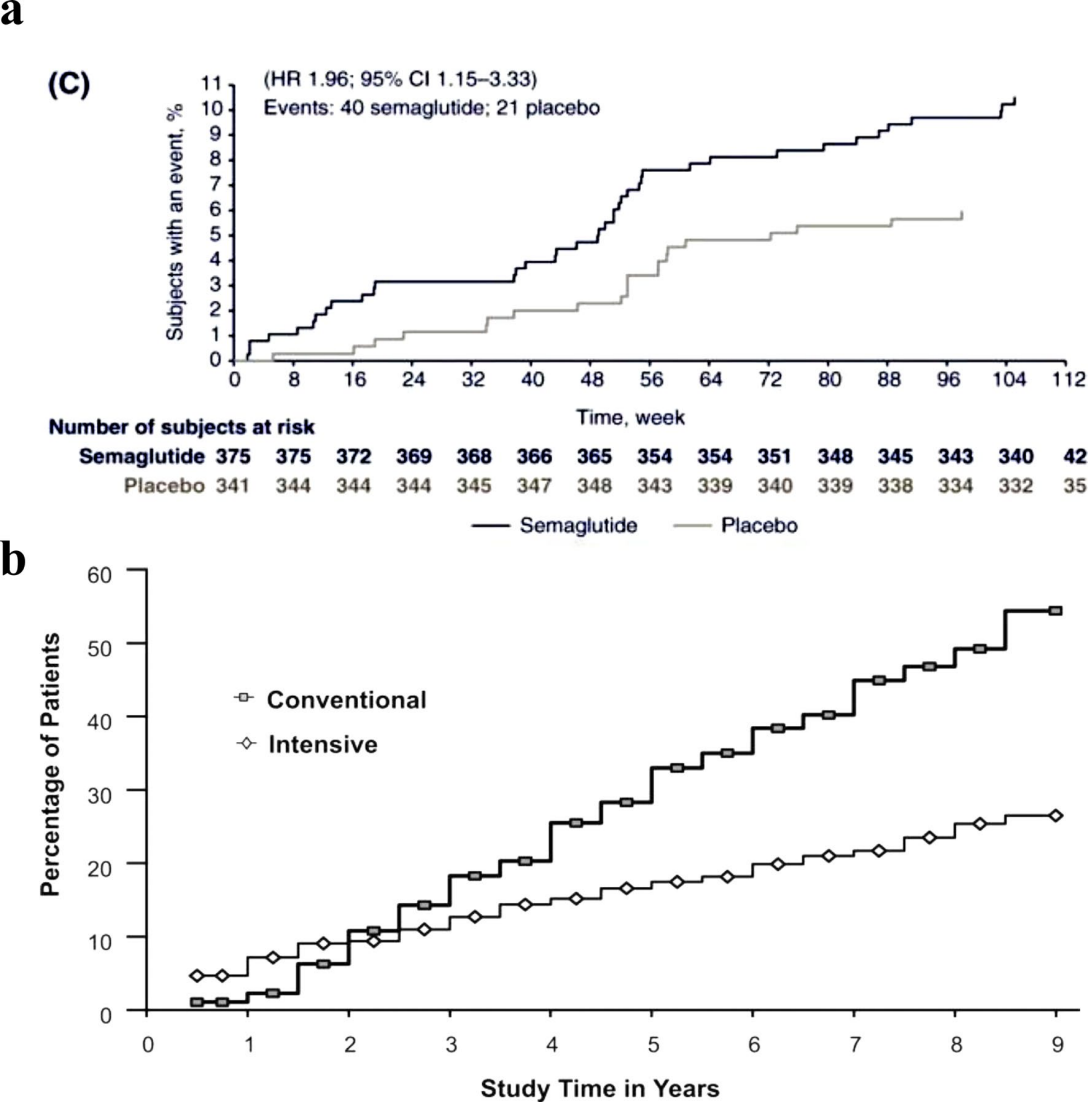
Early worsening of DR progression with semaglutide and insulin. **A**. Worsening DR progression with semaglutide in the SUSTAIN‑6 trial [2, 3]. Among patients with T2D using insulin and with DR at baseline, semaglutide (blue curve) was associated with greater progression of retinopathy from weeks 16 to 96 compared with placebo (gray curve). Reprinted with permission from *John Wiley and Sons* and Vilsbøll T, et al. *Diabetes Obes Metab*. 2018;20 (4):889–897 (original Fig. 1C in reference 3). **B**. Early worsening of DR progression with intensive insulin therapy in the DCCT trial [4]. Intensive insulin therapy (open rhomboid markers) for patients with T1D was associated with greater DR progression during the first 2 years compared with the conventional treatment group (shaded rectangular markers), followed by a 54% reduction in DR risk after a mean follow‑up of 6.5 years. Reprinted with permission from *John Wiley and Sons*, *American Diabetes Association*, and Aiello LP; DCCT/EDIC Research Group. *Diabetes Care.* 2014;37 (1):17–23 (original Fig. 2 in reference 4). Abbreviations: DCCT, Diabetes Control and Complications Trial; DR, diabetic retinopathy; EDIC, Epidemiology of Diabetes Interventions and Complications study; SUSTAIN-6, Semaglutide Unabated Sustainability in Treatment of Type 2 Diabetes; T1D, type 1 diabetes; T2D, type 2 diabetes

Glucagon-like peptide-1 (GLP-1)-based therapies have transformed the management of type 2 diabetes (T2D) and obesity, demonstrating exceptional efficacy in glycemic control and weight reduction [1]. Semaglutide is among the most widely prescribed GLP-1 receptor agonists (GLP-1RA) [1]. Paradoxically, semaglutide therapy for T2D has been linked to worsening diabetic retinopathy (DR) ([2, 3], Fig. 1A), a phenomenon also observed with insulin treatment in both type 1 diabetes (T1D, Fig. 1B) and T2D [4, 5]. In the SUSTAIN-6 trial (Semaglutide Unabated Sustainability in Treatment of Type 2 Diabetes), two years of semaglutide therapy was associated with accelerated DR progression ([2, 3], Fig. 1A), including increased risks of vitreous hemorrhage (VH), need for laser photocoagulation, and DR-related blindness - by approximately 2.5-fold, 1.9-fold, and 3-fold, respectively [2, 3]. In contrast, empagliflozin, a sodium-glucose cotransporter 2 inhibitor (SGLT-2i), has been shown to slow DR progression compared with dipeptidyl peptidase-4 inhibitor (DPP-4i) therapy over an average follow-up of eight months [6]. Similarly, metformin is generally associated with reduced risk and slower DR progression through suppression of hepatic glucose production [7, 8].

Understanding the mechanisms underlying DR worsening with insulin and semaglutide therapy is essential for developing strategies to prevent vision loss. Proposed explanation in literature include rapid hemoglobin A1c (HbA1c) reduction, osmotic stress, decreased nutrient supply, impaired retinal blood flow regulation, and the vascular endothelial growth factor (VEGF)-mediated angiogenic pathway [2, 3, 9]. However, the protective effects of empagliflozin and metformin challenge the plausibility of the first four hypotheses - rapid HbA1c reduction, osmotic stress, nutrient deprivation, and impaired retinal circulation - since both agents similarly influence these factors. Moreover, the VEGF hypothesis does not fully account for the inflammatory component of DR pathogenesis. Up to 40% of diabetic macular edema (DME) cases are refractory to anti-VEGF therapy, yet some respond to corticosteroids, underscoring the critical role of inflammation in DR and DME development [10, 11]. This review therefore aims to critically evaluate evidence supporting the angiogenesis-inflammation hypothesis in DR (Angiogenesis–Inflammation Hypothesis) and to examine the roles of angiogenic growth factors and inflammatory cytokines in the biphasic effects of insulin - early worsening of DR followed by long-term risk reduction. It further explores broader implications of this hypothesis, including potential biphasic effects of semaglutide on DR and strategies to mitigate the transient DR exacerbation associated with insulin and insulin secretagogues.

## Methodology on literature search and selection criteria

A comprehensive literature search was conducted using PubMed, which includes the MEDLINE database, to identify publications related to DR. The search covered articles published between January 1, 1993, and September 30, 2025. Specific search terms included “semaglutide and retinopathy” and “insulin and worsening of retinopathy,” yielding 88 and 189 publications, respectively. To explore theoretical frameworks and mechanisms underlying DR, an additional search using the terms “angiogenesis and inflammation and diabetic retinopathy and reviews” identified 276 publications. All retrieved articles were screened for relevance based on their discussion of factors influencing DR onset, progression and DME formation, the relationship between glycemic control and retinopathy outcomes in both short- and long-term contexts, and the association between glycemic improvement and early or transient worsening of DR. Clinically pertinent publications, as determined by the author, were also included. Because the effects of insulin and semaglutide on DR can change over time and may even act in opposite directions [4], combining results from different studies into a single pooled estimate is unlikely to be biologically meaningful or statistically valid.

## Angiogenic and inflammatory mediators in DR

DR is a neurovascular complication of both T1D and T2D [12]. Its prevalence and progression are strongly related to the duration of diabetes and the level of glycemic control [12]. In DR, early retinal neurodegeneration precedes microvascular damage [13]. The retinal neurovascular unit (NVU) - an integrated system of neurons, glial cells, and vascular components - couples neural activation to blood flow, maintains the blood-retinal barrier (BRB), and regulates inflammation and metabolic homeostasis [13]. Chronic hyperglycemia disrupts the structure and function of the retinal NVU [13]. Since the 1990s, the Angiogenesis–Inflammation Hypothesis has emerged as a central framework for understanding DR, integrating earlier theories - including the biochemical theory of glycosylation, the hemodynamic theory of leukostasis, and the endocrine theory involving growth hormone - with a growing body of evidence on vascular growth factors and inflammatory cytokines [14–19]. Key growth factors implicated in DR include VEGF, angiopoietin-2 (Ang-2), insulin-like growth factor-1 (IGF-1), placental growth factor (PlGF), and platelet-derived growth factor (PDGF) [14–19].

VEGF, the principal mediator of both physiological and pathological angiogenesis, also functions as a vascular permeability factor [14–19]. Ang-2, produced by endothelial cells, contributes to pericyte loss, endothelial damage, and increased vascular permeability - leading to microaneurysm formation and DME [17–19]. Both VEGF and Ang-2 exhibit pro-inflammatory properties by upregulating adhesion molecules that promote leukocyte migration and adhesion to the endothelium, a process known as leukostasis [14–19]. Their expression is enhanced by hyperglycemia and hypoxia, and elevated levels have been detected in the retina and vitreous of diabetic patients, with reductions observed following panretinal photocoagulation (PRP) [19, 20]. Experimental injection of VEGF into the eyes of nonhuman primates induces hallmark features of DR across its stages [21, 22]. The central role of VEGF in DR is further supported by the clinical success of anti-VEGF therapies in treating vision-threatening diabetic retinopathy (VTDR), including DME and proliferative diabetic retinopathy (PDR) [23–26]. Faricimab (Vabysmo), a bi-specific antibody targeting both VEGF and Ang-2, has demonstrated efficacy in treating DME with improved durability compared to anti-VEGF monotherapies [26]. IGF-1 stimulates retinal VEGF and endothelial Ang-2 expression and may contribute to PDR, with elevated vitreous levels observed specifically in the PDR stage [14, 15, 27–29]. Interestingly, although T2D is common among patients with acromegaly, retinopathy remains relatively rare, suggesting that systemic IGF-1 alone is insufficient to drive neovascularization without concurrent retinal hypoxia and VEGF upregulation [30]. PlGF indirectly contributes to DR by enhancing VEGF activity, while PDGF is associated with fibrovascular membrane formation in PDR and tractional retinal detachment (TRD) [18, 31].

**Fig. 2:**
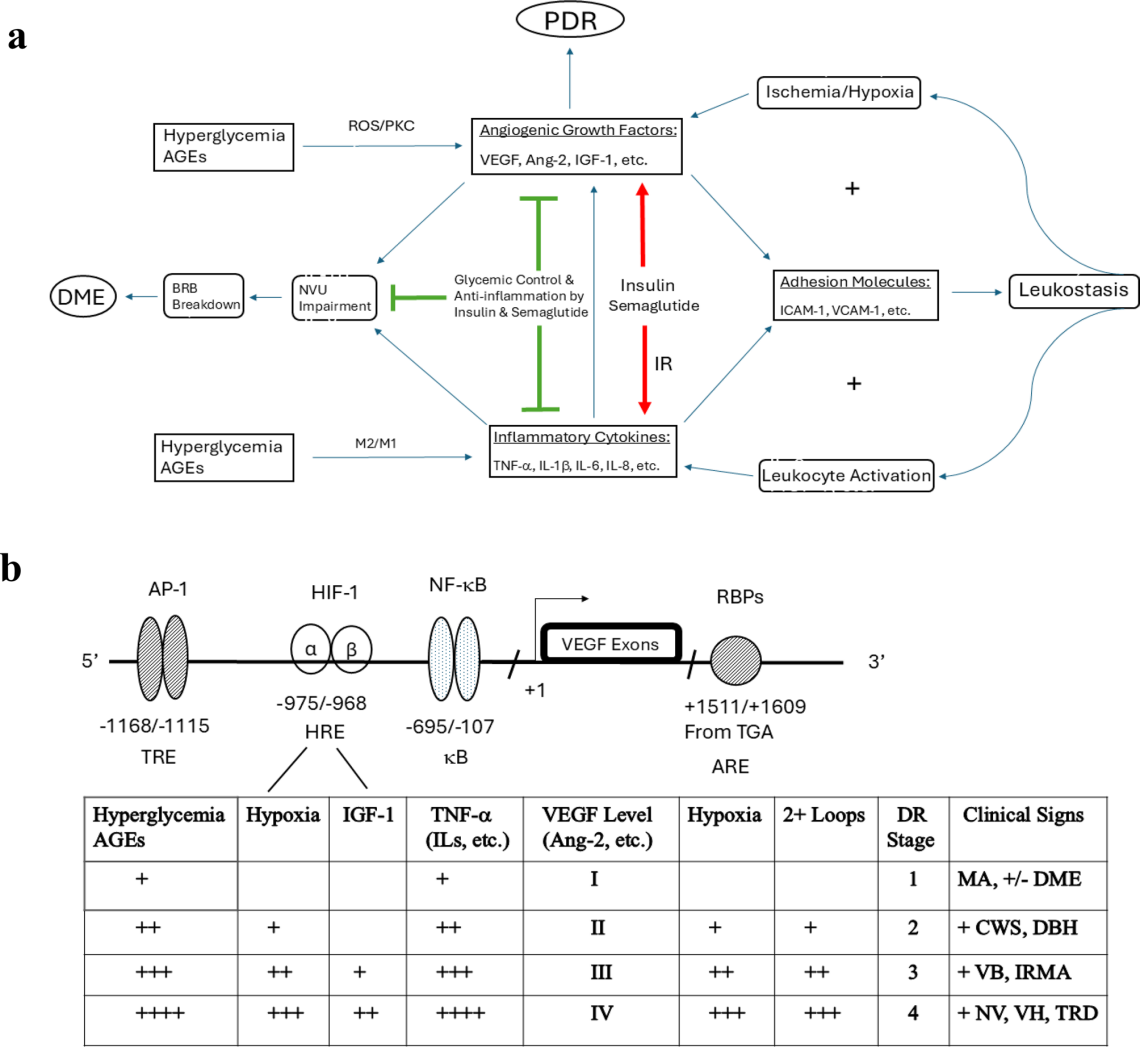
The angiogenesis–inflammation hypothesis in diabetic retinopathy. **A**. The Roles of Angiogenic and Inflammatory Mediators in DR Pathogenesis. Chronic hyperglycemia and the accumulation of AGEs upregulate key angiogenic and inflammatory mediators in the retina—including VEGF, Ang‑2, TNF‑α, and interleukins—leading to NVU dysfunction, pericyte loss, and disruption of endothelial tight junctions. These changes compromise the BRB and promote DME. Inflammatory cytokines further increase VEGF and Ang‑2 levels, while upregulation of ICAM‑1 and VCAM‑1 drives leukostasis, capillary occlusion, and worsening retinal ischemia and hypoxia. These feedback loops amplify VEGF, Ang‑2, TNF‑α, and IL production, and in the presence of IGF‑1, VEGF expression is further enhanced. Once intraocular VEGF and Ang‑2 exceed the angiogenic threshold, neovascularization is triggered, leading to PDR. Semaglutide augments glucose-induced insulin secretion. In the setting of insulin resistance (IR), elevated insulin levels may transiently upregulate retinal VEGF and Ang‑2 and exert pro‑inflammatory effects, potentially contributing to early DR worsening. Over time, sustained glycemic improvement reduces hyperglycemia and AGE formation, improves insulin sensitivity, and favors anti‑inflammatory pathways—including downregulation of TNF‑α and IL‑6—ultimately leading to long‑term reductions in DR risk. Semaglutide also mitigates a broad spectrum of DR risk factors and provides direct retinal neurovascular protection. **B**. A Simplified Schematic of VEGF Gene Regulation and Its Role in DR. Chronic hyperglycemia and the accumulation of AGEs upregulate retinal VEGF, Ang‑2, and TNF‑α through transcriptional pathways involving AP-1 and NF-κB, etc. These mediators disrupt the BRB, causing MA and DME, characteristic of mild NPDR (stage 1). Increased expression of ICAM‑1 and VCAM‑1 by the mediators promotes leukostasis, leading to capillary occlusion, retinal ischemia, and hemorrhages that manifest as CWS and DBH in moderate NPDR (stage 2). Hypoxia further enhances VEGF, Ang‑2, and TNF‑α transcription via HIF‑1 and stabilizes their mRNAs through AU‑rich elements and RNA‑binding proteins. Upregulation of ICAM‑1 and VCAM‑1 drives interconnected hypoxia‑inflammation feedback loops, accelerating angiogenic and inflammatory signaling. These processes worsen vascular leakage and ischemia, progressing to severe NPDR (stage 3), marked by extensive DBH across all quadrants, VB, and IRMA. IGF‑1 and hypoxia act additively to increase VEGF and Ang‑2 expression. When their intraocular levels exceed the angiogenic threshold, NVD and NVE develop, defining PDR (stage 4). In advanced PDR, severe ischemia enables diffusible VEGF₁₂₁, semi‑diffusible VEGF₁₆₅, Ang‑2, and TNF‑α to reach the anterior chamber and drive neovascularization of the iris and angle (NVG). Recurrent VH, together with PDGF‑mediated fibrovascular proliferation, contributes to epiretinal fibrovascular membrane formation and TRD. Adapted from Lu M, 1999 [14]. Abbreviations: 2+ Loops, the 2 interlinked positive feedback loops involving hypoxia and inflammation in diabetic retinopathy; AGEs, advanced glycation endproducts; Ang-2, angiopoietin-2; AP-1, activator protein-1; ARE: AU-rich elements; bent arrow, transcription start site; BRB, blood-retinal barrier; CWS, cotton-wool spot; DBH, dot and blot hemorrhage; DME, diabetic macular edema; DR, diabetic retinopathy; HRE, hypoxia-responsive element; ICAM-1, intercellular adhesion molecule-1; IGF-1, insulin-like growth factor-1; IL, interleukin; IRMA, intraretinal microvascular abnormality; κB, NF-κB-binding site; M2/M1, macrophage polarization from the anti-inflammatory M2 phenotype toward the pro-inflammatory M1 phenotype; MA, microaneurym; NVD, neovascularization of the optic disc; NVE, neovascularization elsewhere retina; NVG, neovascular glaucoma; NVU, neurovascular unit; PDR, proliferative diabetic retinopathy; PKC, protein kinase C; RBPs, RNA-binding proteins; ROS, reactive oxygen species; TGA, transcription stop codon TGA; TNF-α, tumor necrosis factor-alpha; TRD, tractional retinal detachment; TRE, TPA response element; UTR, mRNA 3’ untranslated region; VB, venous beading; VCAM-1, vascular cell adhesion molecule-1; VEGF, vascular endothelial growth factor; VH, vitreous hemorrhage

Although DR is primarily a neurovascular disorder, inflammation plays a significant role in its pathogenesis [11, 16–18]. Macrophage polarization is a key contributor to this process [32]. Under conditions of chronic hyperglycemia, oxidative stress, and accumulation of advanced glycation end-products (AGEs), retinal macrophages and microglia predominantly shift toward a pro-inflammatory M1 phenotype. These M1 macrophages secrete cytokines such as tumor necrosis factor-alpha (TNF-α) and interleukins (IL-1β, IL-6, IL-8, etc.), which contribute to endothelial dysfunction and disruption of the BRB [32]. As DR progresses, a transition toward the anti-inflammatory M2 phenotype occurs, characterized by the release of IL-10 and VEGF [32]. While M2 macrophages promote inflammation resolution and tissue repair, their proangiogenic activity may also drive pathological neovascularization in PDR. The imbalance between M1 and M2 polarization thus underlies both vascular and inflammatory components of DR [32]. Elevated levels of TNF-α, IL-1β, IL-6, and IL-8 have been detected in the serum and vitreous of patients with DR [16–18, 33]. In addition, intercellular adhesion molecule-1 (ICAM-1) and vascular cell adhesion molecule-1 (VCAM-1) are upregulated in DR, with their expression correlating with disease severity [16–18]. These cytokines and adhesion molecules act synergistically to promote leukostasis and enhance reactive oxygen species (ROS) production, leading to apoptosis of endothelial cells and pericytes [16–18, 34, 35]. They also disrupt endothelial tight junctions, further compromising the BRB. TNF-α has been shown to upregulate both VEGF and Ang-2 expression, while IL-6 regulates TNF-α production in DR [36, 37]. This inflammatory cascade creates a positive feedback loop that amplifies retinal inflammation (Fig. 2A). Blockades of VEGF, Ang-2, ICAM-1, or VCAM-1 in experimental animals reduced diabetic retinal leukostasis and vascular leakage [34, 35].

DR affects not only the retina but also the vitreous. Diabetes induces progressive vitreous changes through inflammation, altered vitreoretinal adhesion, and ischemia-driven neovascularization, leading to vision-threatening complications [38]. The vitreous serves as a reservoir for angiogenic and inflammatory mediators, provides a scaffold for neovascular growth, and acts as a mechanical driver of traction. Diabetes delays physiologic posterior vitreous detachment by reducing vitreous liquefaction and reinforcing vitreoretinal adhesion through glycation‑induced collagen cross‑linking, extracellular matrix accumulation, inflammation, and oxidative stress [38]. The resulting stronger vitreoretinal adhesion—particularly at the macula and at sites of neovascularization—predisposes patients to DME, VH, and TRD. Chronic hyperglycemia increases the likelihood of vitreomacular traction and epiretinal membrane formation, contributing to the tractional component of DME. These factors can reduce the efficacy of anti‑VEGF therapy and may ultimately necessitate vitrectomy in refractory cases [38]. Vitrectomy removes angiogenic and inflammatory mediators from vitreous, eliminates the scaffold for neovascularization, releases the vitreoretinal traction, and improves retinal oxygenation. This procedure not only clears VH but also improves DME and reduces recurrence of PDR [38].

## The angiogenesis–inflammation hypothesis in diabetic retinopathy 

**Fig. 3:**
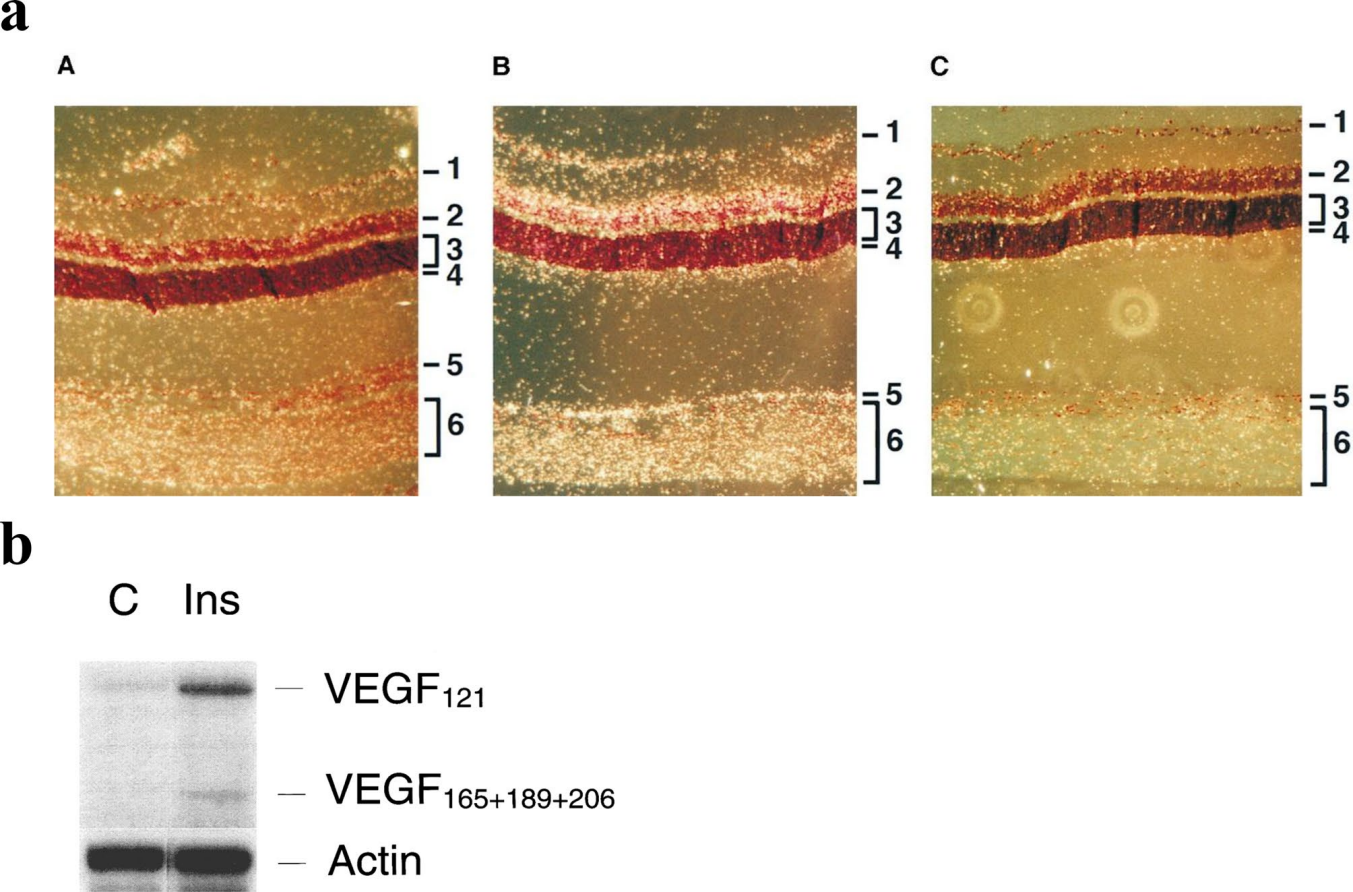
AGEs and insulin enhance retinal VEGF expression. **A**. AGEs Increase Rat Retinal VEGF mRNA in vivo. AGEs increased VEGF mRNA (**B**) in the ganglion cell layer (1), the inner nuclear layer (2), and the RPE layer (5), compared with the control BSA-injected eye (**A**) and sense probe used (**C**). Reprinted with permission from *American Society for Clinical Investigation* and Lu M, et al., 1998 [45]. **B**. Insulin Increases Retinal Cell VEGF mRNA in vitro. Insulin increased VEGF mRNA isoforms VEGF_121_, VEGF_165_, VEGF_189_ and VEGF_206_ in human RPE cells. RNase protection assays were performed using β-Actin RNA for quantification calibration. C, control; Ins, insulin. Reprinted with permission from *Association for Research in Vision & Ophthalmology (ARVO)* and Lu M, et al., 1999 [46]. Abbreviations: AGEs, advanced glycation endproducts; BSA, bovine serum albumin; C, control; Ins, insulin; RPE, retinal pigment epithelium; VEGF, vascular endothelial growth factor

The Angiogenesis–Inflammation Hypothesis suggests that hyperglycemia promotes DR progression and DME formation by upregulating angiogenic growth factors and inflammatory cytokines, which act synergistically on the retinal NVU [14–19]. Although VEGF, Ang-2, and TNF-α are structurally unrelated, they are all upregulated in the retina in response to hypoxia, hyperglycemia, AGEs, and inflammatory stimuli [14–19]. Their gene promoters share key cis-regulatory elements recognized by transcription factors such as hypoxia-inducible factor-1 (HIF-1), nuclear factor kappa-light-chain-enhancer of activated B cells (NF-κB), specificity protein 1 (SP1), and activator protein 1 (AP-1). Additionally, their mRNAs contain AU-rich elements (AREs) in the 3′ untranslated regions (3′ UTRs), which regulate transcript stability through interactions with RNA-binding proteins (RBPs) [14, 15, 39–42]. Figure 2B presents a simplified schematic of VEGF gene regulation and its role in DR [14, 15]. Hyperglycemia and AGEs upregulate retinal expressions of VEGF, Ang-2, and TNF-α through the generation of ROS and activation of protein kinase C (PKC) [14–19, 43–45]. These pathways subsequently activate transcription factors such as AP-1, NF-κB, and SP1, which promote transcription of the respective mediator genes [14–19, 43–45]. Figure 3A illustrates that AGEs increase retinal VEGF expression [45].

**Fig. 4:**
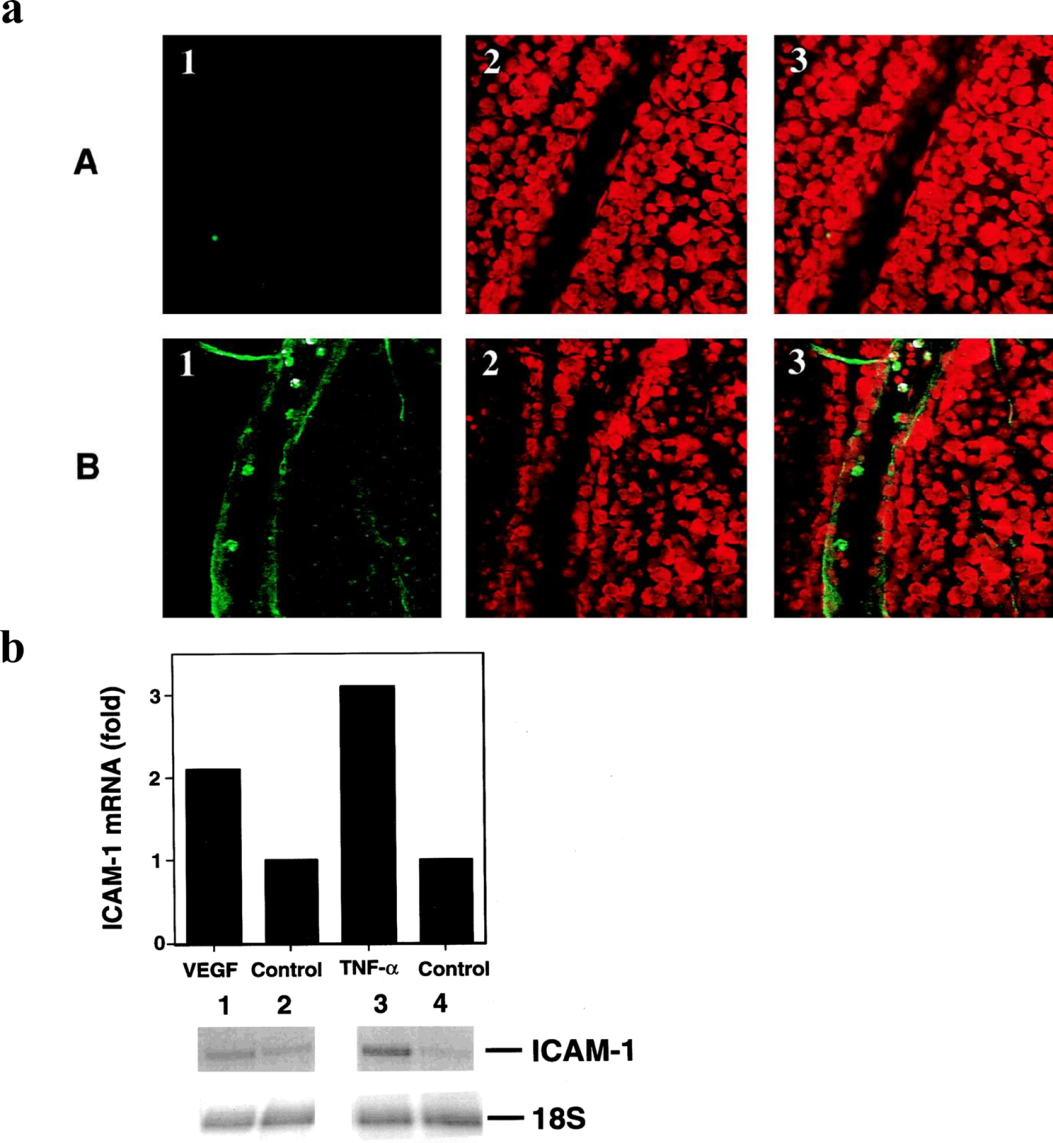
VEGF and TNF-α Increase Retinal ICAM-1 Expression. **A.** VEGF Increases ICAM-1 Immunostaining in Mouse Retinal Vasculature. VEGF-injected eyes (B) showed a marked upregulation of ICAM-1 protein in the retinal post-capillary venules and veins (green stain), compared with the control PBS-injected eyes (A). **B.** VEGF and TNF-α increase rat retinal ICAM-1 mRNA after intravitreal injection. RNase protection assays were performed using 18S RNA for quantification calibration. Reprinted with permission from Association for Research in Vision & Ophthalmology (ARVO) and Lu M, et al., Invest Ophthalmol Vis Sci. 1999;40(8):1808-1812 [47]. Abbreviations: ICAM-1, intercellular adhesion molecule-1; PBS, phosphate buffered saline; TNF-α, tumor necrosis factor-alpha; VEGF, vascular endothelial growth factor

Elevated levels of VEGF, Ang-2, and TNF-α compromise the retinal NVU and disrupt the BRB, leading to microaneurysms (MA) and DME formation, hallmarks of mild non-proliferative DR (NPDR, stage 1). These factors also upregulate ICAM-1 and VCAM-1 expression in retinal vasculature [14, 15, 34, 35, 47]. Figures 4A and B show that VEGF and TNF-α enhance retinal ICAM-1 production [47]. This promotes leukostasis, leading to capillary occlusion, retinal ischemia, and hemorrhages, which manifest as cotton-wool spots (CWS) and dot/blot hemorrhages (DBH) in moderate NPDR (stage 2). Hypoxia is a key driver of VEGF, Ang-2, and TNF-α transcription via HIF-1, and enhances mRNA stability post-transcriptionally through AREs and RBPs in the 3′ UTR [14, 15, 40, 42]. Upregulation of ICAM-1 and VCAM-1 activates the two interlinked positive feedback loops involving hypoxia and inflammation, accelerating the production of angiogenic and inflammatory mediators (Fig. 2A). This exacerbates retinal leakage and ischemia, leading to severe NPDR (stage 3), characterized by extensive DBH across all four quadrants, venous beading (VB), and intraretinal microvascular abnormalities (IRMA).

Furthermore, IGF-1 and hypoxia additively enhance VEGF and Ang-2 expression [14, 15, 27, 28]. When their levels surpass the angiogenic threshold, neovascularization of the optic disc (NVD) and elsewhere in the retina (NVE) occurs, marking the onset of PDR (stage 4). In advanced PDR, escalating retinal ischemia and hypoxia enable diffusible mediators - such as Ang-2, TNF-α, VEGF₁₂₁, and the semi-diffusible VEGF₁₆₅ isoform - to migrate anteriorly down the vitreal gradient into the anterior chamber, where they drive neovascularization of the iris and angle, a hallmark of neovascular glaucoma (NVG). Recurrent VH in PDR, combined with PDGF activity, contributes to epiretinal fibrovascular membrane formation and TRD [18, 31].

While angiogenic growth factors play a central role in DR progression, inflammatory cytokines appear to be the key drivers of DME formation. DME can develop at any stage of DR. Although its prevalence increases with more advanced stages of DR, the severity of DME does not necessarily correlate with the severity of retinopathy. DME primarily results from the breakdown of BRB, whereas DR staging reflects capillary nonperfusion, ischemia, and neovascularization [14–18]. This distinction is supported by clinical observations showing that some cases of DME resistant to anti-VEGF therapy still respond to corticosteroids [10, 11]. Nevertheless, DME and DR are interconnected processes, with hyperglycemia being the principal risk factor in both T1D and T2D. In the Diabetes Control and Complications Trial (DCCT) involving patients with T1D, intensive insulin therapy over 6.5 years reduced the risk of developing DR by 76%, slowed its progression by 54%, and lowered the incidence of DME by 26%, corresponding to an average 2% reduction in HbA1c [4]. Similarly, in the UK Prospective Diabetes Study (UKPDS), six years of intensive therapy with insulin, sulfonylureas (SU), or metformin in patients with T2D reduced the risk of retinopathy, yielding an approximately 31% risk reduction for every 1% decrease in HbA1c [48].

## Insulin increases angiogenic and inflammatory mediators in retina

The early worsening of DR has been consistently reported with the use of insulin and insulin secretagogues such as SUs and semaglutide [2–5, 9, 49], but not with insulin-independent antidiabetic agents like metformin or empagliflozin [6–8, 49]. These findings suggest that insulin itself may play a role in transient DR exacerbation. Experimental studies have demonstrated that insulin increases retinal expression of VEGF and endothelial Ang-2 [46, 50, 51]. As illustrated in Fig. 3B, insulin upregulates retinal VEGF gene expression [46].

Additionally, acute intensive insulin therapy has been shown to exacerbate breakdown of the BRB, an effect that can be mitigated by anti-VEGF treatment [50]. Insulin also promotes Ang-2 expression in human umbilical vein endothelial cells through a c-Fos–mediated transcriptional pathway [51]. In insulin-resistant states such as T2D and obesity, hyperinsulinemia contributes to macrophage polarization from the anti-inflammatory M2 phenotype toward the pro-inflammatory M1 phenotype [32, 52, 53]. This shift drives inflammation and endothelial dysfunction via activation of NF-κB and increased production of pro-inflammatory cytokines including TNF-α, IL-1β, and IL-6 [32, 52, 53].

The interplay between insulin and hyperglycemia in modulating the angiogenic and inflammatory mediators may help explain the biphasic effects of insulin therapy on DR - early worsening followed by long-term risk reduction [4, 5]. The early worsening is potentially mediated by insulin-induced upregulation of VEGF and Ang-2, along with the pro-inflammatory effects of hyperinsulinemia in the context of insulin resistance (Fig. 2A). During the initial phase of treatment, these adverse effects on retinal NVU may outweigh the benefits of improved glycemic control achieved through insulin therapy. However, with long-term insulin use, sustained glycemic control reduces the duration of hyperglycemia and the formation of AGEs. This, in turn, alleviates glucose toxicity, supports pancreatic β-cell recovery, suppresses lipotoxicity in muscle and liver by lowering circulating fatty acid levels, and enhances insulin receptor function by mitigating inflammation and oxidative stress [32, 52, 53]. As insulin resistance improves, insulin can exert anti-inflammatory effects by downregulating pro-inflammatory cytokines such as TNF-α and IL-6, while promoting anti-inflammatory cytokines like IL-10 [52, 53]. Additionally, the insulin doses required for glycemic control may decrease in some patients, thereby reducing retinal angiogenic activity and inflammation. Over time, these beneficial effects from sustained glycemic control may surpass the initial retinal harm, ultimately contributing to a reduction in long-term DR risk [4, 54].

## GLP-1 augments glucose-induced insulin secretion

All GLP-1RAs are insulin secretagogues [1]. GLP-1 enhances insulin secretion in a glucose-dependent manner [1, 55, 56], and semaglutide may contribute to DR worsening through mechanisms similar to those of insulin therapy. In response to oral nutrient intake, the intestine releases incretin hormones - GLP-1 and glucose-dependent insulinotropic polypeptide (GIP) - which together account for up to 66% of postprandial insulin secretion [57]. This phenomenon, known as the incretin effect, diminishes proportionally with increasing HbA1c and body mass index (BMI) [1, 57]. DPP-4is prevent the enzymatic degradation of endogenous GLP-1 and GIP and are therefore considered indirect insulin secretagogues [58, 59].

**Fig. 5:**
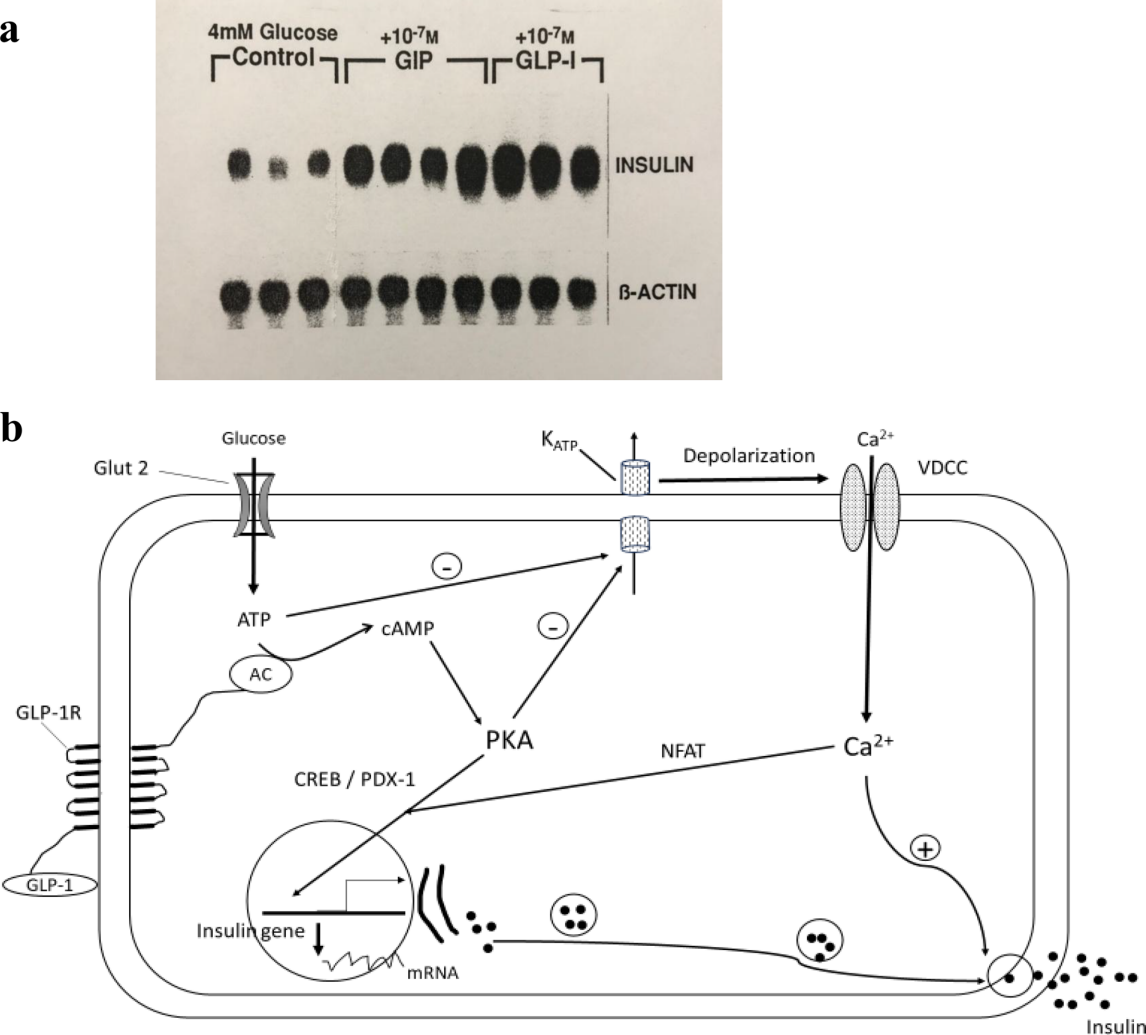
GLP-1 enhances insulin secretion and insulin gene expression. **A**. GLP-1 and GIP Increase Insulin mRNA in a Pancreatic β-cell Line. Both GLP-1 and GIP increase the insulin mRNA level from a transformed β-cell line (HIT). Northern blot analysis was performed using β-Actin as control. From Lu M, 1993 [55], and reprinted with permission from *Association of American Medical Colleges (AAMC)* and Lu M et al., 1993 [63]. **B**. Mechanism of GLP-1–Induced Insulin Secretion and Gene Expression. GLP‑1 binds to the GLP‑1 receptor (GLP‑1R) on pancreatic β‑cells, activating adenylate cyclase (AC) and increasing intracellular cyclic AMP (cAMP). Elevated cAMP activates protein kinase A (PKA), which phosphorylates ATP‑sensitive potassium channels (K-ATP), enhancing their closure in response to ATP generated from glucose metabolism. This results in membrane depolarization, opening of voltage‑dependent calcium channels (VDCCs), and a subsequent rise in cytosolic calcium that triggers insulin secretion. Beyond stimulating exocytosis, calcium influx and PKA activation promote insulin gene transcription through key transcription factors, including cAMP response element–binding protein (CREB), pancreatic and duodenal homeobox 1 (PDX‑1), and nuclear factor of activated T cells (NFAT). These pathways enhance insulin biosynthesis and replenish intracellular insulin stores. Adapted from Lu M, 1993 [55]. Abbreviations: AC, adenylyl cyclase; Ca^2+^, calcium ion; CREB, cAMP responsive element binding protein; GIP, glucose-dependent insulinotropic polypeptide; GLP-1, glucagon-like peptide-1; GLP-1R, glucagon-like peptide-1 receptor; Glut-2, glucose transporter 2; K-ATP, ATP-sensitive potassium channel; M, molar; mM, millimolar; NFAT, nuclear factor of activated T-cells; PDX-1, pancreatic duodenal homeobox 1; PKA, protein kinase A; VDCC, voltage-dependent calcium channel

GLP-1 binds to its receptor (GLP-1R) on pancreatic β-cell membranes, enhancing insulin secretion and gene expression via cyclic AMP (cAMP) and calcium-mediated signaling pathways [55, 56, 60–62]. As shown in Fig. 5A, both GLP-1 and GIP increase insulin mRNA expression in a pancreatic β-cell line [55, 63]. A simplified signaling pathway for GLP-1 action in pancreatic β-cells is depicted in Fig. 5B [55]. This 1993 model has been validated and expanded over the past three decades, with additional mechanisms extensively reviewed [61]. Similarly, GIP enhances insulin secretion and gene expression through analogous mechanisms after binding to its own receptor on pancreatic β-cells [63]. By concurrently activating both GLP‑1 and GIP receptors, the dual incretin receptor agonist tirzepatide has demonstrated the greatest therapeutic efficacy to date in the treatment of T2D and obesity. In clinical trials, tirzepatide lowered HbA1c by up to 2.58% and produced weight loss as much as 24.5% [1, 64]. In comparison, semaglutide - acting solely through the GLP‑1 receptor - reduced HbA1c by up to 2.2% and body weight by as much as 15% [1, 65]. Based on UKPDS data demonstrating that each 1% decrease in HbA1c corresponds to an approximately 31% reduction in DR risk [48, 54], these glycemic improvements would translate to an estimated 80% reduction with tirzepatide and 68% with semaglutide. 

## Systemic and Retinal Effects of GLP-1

Beyond glycemic control via insulinotropic effects, GLP-1RAs exert multiple systemic benefits. They suppress appetite and glucagon secretion, slow gastrointestinal motility, and reduce inflammation, contributing to weight loss, improved lipid profiles, and normalized blood pressure [1]. These actions collectively lower cardiovascular, renal, and hepatic complications associated with obesity and type 2 diabetes [1, 66, 67]. In the retina, GLP-1 mitigates neurodegeneration by inhibiting retinal ganglion cell apoptosis, reducing glial cell activation and inflammatory cytokine release, and alleviating hypoxia through enhanced endothelial nitric oxide (NO) availability [66, 67]. Table 1 summarizes the systemic and retinal effects of GLP-1. The anti-inflammatory effects of GLP-1RAs are both direct - via GLP-1 receptor activation on Müller glia and microglia - and indirect, through improvements in weight and glycemia [1, 66–71]. GLP-1 inhibits NF-κB, a central transcription factor in inflammation, leading to reduced levels of TNF-α, IL-1β, and IL-6 [1, 66–71]. It also reduces ROS, promotes anti‑inflammatory macrophage polarization, and provides antioxidative and cytoprotective effects in retinal endothelial cells [66–71]. A meta-analysis of 52 randomized controlled trials (RCT) demonstrated that GLP-1RAs significantly reduced TNF-α, IL-1β, and IL-6 levels in patients with T2D compared to conventional therapies involving insulin and oral agents [69]. Preclinical studies have shown that semaglutide attenuates DR progression by ameliorating retinal vasculopathy and oxidative stress in both in vivo and in vitro models, likely through mechanisms involving reduced inflammation and apoptosis [70, 71].

**Fig. 6:**
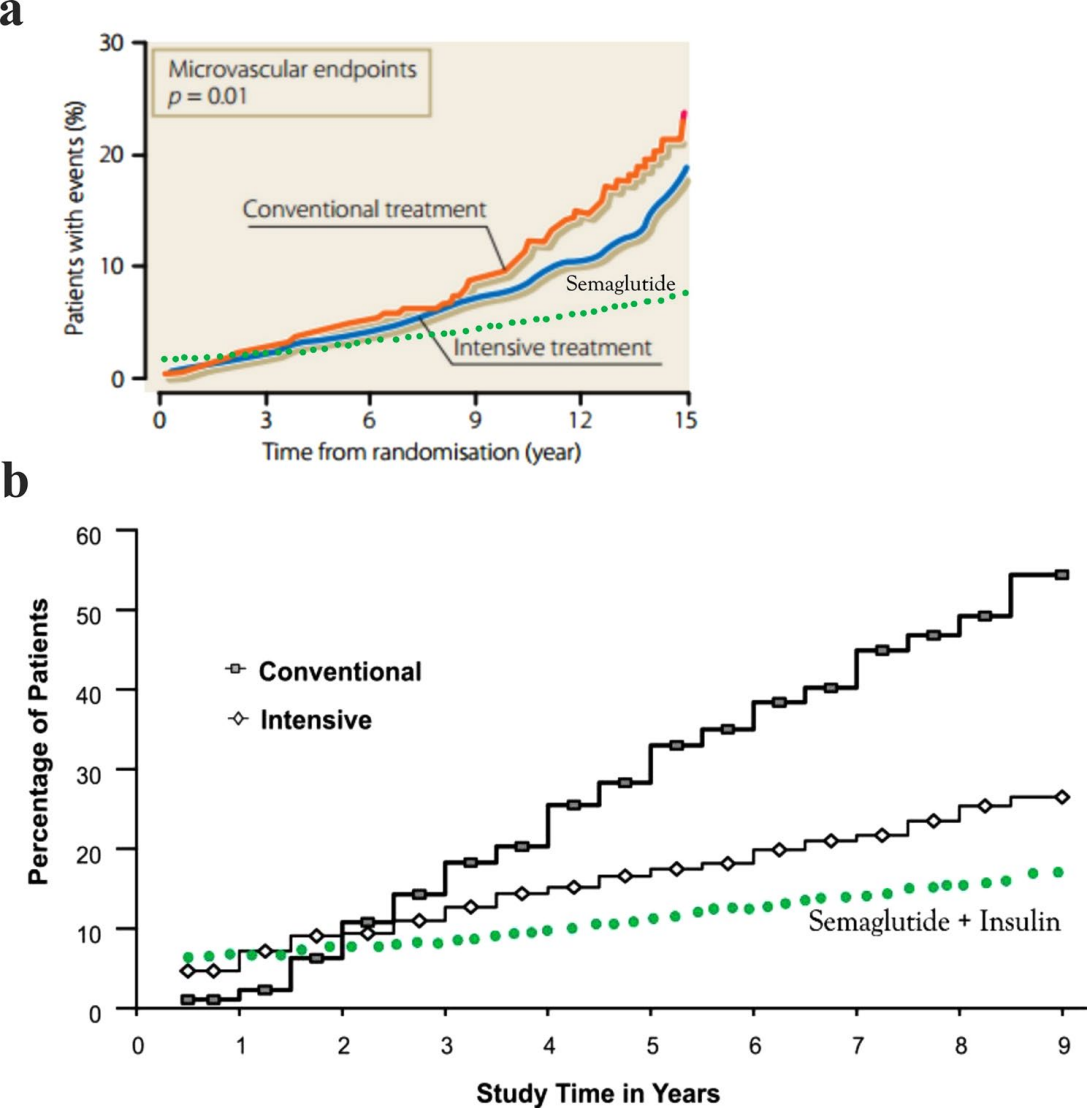
Estimated biphasic effects of semaglutide on DR progression. **A**. Estimated biphasic effects of semaglutide on DR progression in T2D. The predicted DR‑progression trajectory with semaglutide therapy is superimposed on the original UKPDS 33 microvascular event curves. The orange and blue curves show the percentages of patients developing microvascular complications under conventional and intensive therapy, respectively; the intensive arm received insulin or sulfonylureas. Early worsening associated with semaglutide is illustrated using green dots for the first 2 years, based on findings from SUSTAIN‑6 trial. Beyond this period, semaglutide is projected to confer an approximate 68% reduction in long‑term DR risk after a mean follow‑up of 6 years (as calculated in the text). Figure reconstructed with permission from *Elsevier* and UKPDS Group, *Lancet*. 1998;352:837–853 [54]. **B**. Estimated biphasic effects of semaglutide add‑on therapy on DR progression in T1D. The predicted DR‑progression trajectory with semaglutide added to insulin therapy (green dots) is superimposed on the original DCCT curves. Early worsening associated with semaglutide add‑on therapy is depicted with green dots during the first 2 years, followed by an estimated additional 16% reduction in long‑term DR risk compared with the intensive treatment group after a mean follow‑up of 6.5 years (calculation provided in the text). Figure reconstructed with permission from *John Wiley and Sons*, *American Diabetes Association*, and Aiello LP; DCCT/EDIC Research Group. *Diabetes Care.* 2014;37 (1):17–23 [4]

As shown in Table 2, semaglutide addresses a broader spectrum of systemic DR risk factors than insulin, including hypertension (HTN), dyslipidemia, chronic kidney disease (CKD), obesity, and obstructive sleep apnea (OSA). HTN promotes retinal VEGF expression via angiotensin II (ANG II) and activation of PKC/phosphoinositide 3-kinase (PI3K) pathways [72, 73]. In the UKPDS, every 10-mmHg reduction in systolic blood pressure (SBP) was associated with an 11% decrease in the need for photocoagulation or treatment of VH [48]. Dyslipidemia elevates retinal TNF-α, IL-1β, and IL-6 through oxidized low-density lipoprotein (ox-LDL) and increased ROS, activating NF-κB signaling. Elevated triglyceride and LDL concentrations contribute to retinal lipid accumulation, leading to hard exudate formation and exacerbation of DME [12, 74]. CKD exacerbates DR through ANG II–mediated upregulation of angiogenic and inflammatory mediators [75]. Obesity drives hyperinsulinemia and insulin resistance (IR), increasing risks of hyperglycemia, HTN, dyslipidemia, CKD, inflammation, oxidative stress, and OSA [76]. OSA may increase DR risk by upregulating VEGF and Ang-2 during nocturnal hypoxia [77]. Weight reduction through bariatric surgery or pharmacotherapy has been associated with lower DR risk [78]. Semaglutide and tirzepatide are currently the most effective pharmacologic options for weight loss, achieving up to 15% and 24.5% reductions in body weight, respectively [1]. This substantial weight loss mitigates multiple major DR risk factors in Table 2. Semaglutide significantly reduced SBP and improved dyslipidemia, and its overall antihypertensive effect may be even greater, as patients in the trials frequently had their antihypertensive medications tapered [79, 80]. Based on combined effects of HbA1c reduction of 2.2% and SBP improvement of 5-mmHg, long-term semaglutide therapy is predicted to reduce DR risk by at least 68%, a hypothesis under investigation in the ongoing FOCUS trial (NCT03811561), initiated in 2019 and projected to conclude in 2027 [81]. Figure 6A illustrates an estimated DR risk curve for semaglutide in T2D, superimposed on UKPDS data [54], with early worsening depicted according to findings from the SUSTAIN-6 trial [2, 3]. UKPDS did not clearly capture early worsening because participants generally had minimal or no baseline retinopathy, experienced slower and smaller reductions in HbA1c (approximately 1% in UKPDS versus 2% in DCCT), and underwent less frequent retinal evaluations (typically annually or longer), limiting the detection of this transient phenomenon observed in the DCCT [4, 54]. Both semaglutide and tirzepatide have been used as add‑on therapies to insulin in patients with T1D and obesity, producing additional HbA1c reductions of up to 0.54% and 0.68%, respectively [82, 83]. Based on DCCT data indicating that each 1% decrease in HbA1c yields roughly a 30% reduction in DR risk [4], these glycemic improvements correspond to approximately 16% and 20% incremental decreases in DR risk beyond the effect of insulin therapy alone, as illustrated in Figure 6B. 

## Controversies in the retinopathy risk of semaglutide

The conflicting reports regarding the risk of DR associated with semaglutide [2, 3, 9, 84–86] may be attributed to differences in treatment duration and comparator agents used across studies. Like insulin therapy in the DCCT trial [4], semaglutide may exhibit a biphasic effect on DR due to its insulinotropic action: short-term treatment (1-2 years) may lead to worsening [2, 3]; intermediate duration (3-4 years) may show a neutral effect [84]; and long-term therapy (≥6 years) may confer protection [85, 86]. A Swedish observational study demonstrated that GLP-1RAs significantly reduced the incidence of DR [85]. Furthermore, a recent retrospective cohort study found that GLP-1RAs were associated with a lower risk of VH, NVG, and blindness [86]. The UKPDS demonstrated that insulin, insulin secretagogue SUs, and insulin-independent glucose-lowering agent metformin all reduce DR risk after six years of treatment [48, 54]. SUs stimulate insulin secretion through a glucose-independent mechanism by directly closing the ATP-sensitive potassium channels on the pancreatic β-cell membrane [87, 88]. Although SUs have been linked to DR progression [9, 49], long-term SU use has demonstrated a reduction in DR risk over 6 to 10 years [54, 87], with each year of SU therapy associated with a 7% decrease in DR risk [87]. Likewise, DPP-4is have shown increased DR risk after one year of use [58], but a reduction after a decade of therapy [59], reflecting a biphasic pattern like insulin. Given that hyperglycemia is the primary risk factor for DR, all glucose-lowering agents are expected to provide protective effects with long-term use, including semaglutide and tirzepatide. 

**Fig. 7:**
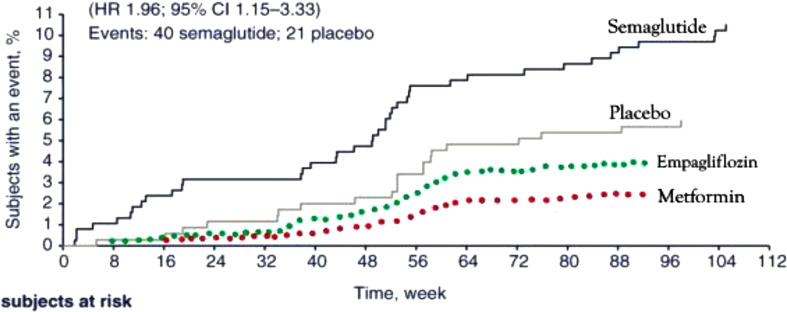
Predicted effects of empagliflozin and metformin on DR progression in T2D. For patients with T2D and DR at baseline, empagliflozin and metformin are predicted to reduce the risk of DR progression between weeks 16 and 96 compared with placebo (gray curve), without the early worsening observed with semaglutide (blue curve). The estimated risk reductions are 25% for empagliflozin (green dotted curve) and 56% for metformin (red dotted curve), as detailed in the text. Figure reconstructed with permission from *John Wiley and Sons* and Vilsbøll T, et al. *Diabetes Obes Metab*. 2018;20 (4):889–897 [3]

The choice of comparator - whether insulin, an insulin secretagogue, or an insulin-independent agent such as empagliflozin or metformin can significantly influence conclusions regarding the DR risk of a test agent. The maximal HbA1c-lowering effects of empagliflozin and metformin have been reported as 0.8% and 1.8%, respectively [88], which correspond to estimated reductions in DR risk of about 25% and 56% based on the UKPDS finding that each 1% decrease in HbA1c reduces DR risk by 31% [48, 54]. As illustrated in Fig. 7, the apparent DR risk of semaglutide may be overestimated when compared with empagliflozin or metformin. Conversely, the protective effect of empagliflozin and metformin may be overestimated when insulin or semaglutide is used as the comparator, due to their association with early DR worsening. When SGLT-2is were added to metformin therapy, an increase rather than a reduction in DR risk was observed [89], likely because metformin itself provides more than twice the estimated protective effect - approximately a 56% risk reduction compared with 25% for SGLT-2is (Figure 7). Table 3 summarizes the conflicting reports on DR risks associated with insulin and insulin secretagogues, along with potential explanations based on the Angiogenesis–Inflammation Hypothesis.

**Table 1:** Systemic and retinal effects of GLP-1

	Systemic Target Organ	GLP-1 Action	Clinical Effect	References
1	Pancreatic β-cell	Augment glucose-induced insulin secretion	Glycemic control	[1, 55, 56, 60, 61]
2	Pancreatic α-cell	Suppress glucagon secretion	Glycemic control	[1, 61]
3	CNS Hypothalamus	Decrease appetite	Weight loss	[1, 61]
4	Stomach	Delay gastric emptying	Weight loss	[1, 61]
	**Direct Retinal Site**			
5	Retinal ganglion cell	Decrease ganglion cell apoptosis	Protect retinal NVU	[66, 67]
6	Müller glia, microglia	Suppress NF-κB signaling	Reduce leukostasis, decrease TNF-α, IL-1β, IL-6, etc.	[68, 69]
7	Endothelial cell	Antioxidative and cytoprotective, enhance NO availability and reduce capillary constriction	Improve hypoxia	[70, 71]

Abbreviations: CNS, central nervous system; GLP-1, glucagon-like peptide-1; IL, interleukin; NF-κB, nuclear factor kappa-light-chain-enhancer of activated B cells; NO, nitric oxide; NVU, neurovascular unit; TNF-α, tumor necrosis factor-alpha

Explained in text Section “Systemic and retinal effects of GLP-1”.

**Table 2:** Effects of insulin and semaglutide on DR risk factors

	DR Risk Factor	Explanation based on the Angiogenesis-Inflammation Hypothesis	Insulin	Semaglutide	References
1	Hyperglycemia	Hyperglycemia and AGEs increase VEGF, Ang-2, TNF-α, and related mediators through ROS and PKC–mediated activation of AP‑1, SP1, and NF-κB	+	+	[1, 65]
2	Hypertension	HTN increases VEGF through ANG II and PKC/PI3K that activate HIF-1, NF-κB, AP-1, and other transcriptional regulators	o	**+**	[72, 73, 79, 80]
3	Dyslipidemia	Dyslipidemia increases TNF-α, IL-1β and IL-6, and related cytokines through ox-LDL-induced ROS production and enhanced NF-κB signaling. Elevated TG and LDL exacerbate DME by promoting hard exudate formation.	o**+**	**+**	[74, 79]
4	CKD	CKD increases VEGF, Ang-2, TNF-α, and related mediators via ANG II–mediated activation of HIF-1, NF-κB, AP-1, and other pathways	o**+**	**+**	[1, 73, 75]
5	Obesity	Obesity increases VEGF, Ang-2, TNF-α, and related mediators through hyperinsulinemia in the IR state, along with worsening hyperglycemia, elevated SBP, dyslipidemia, CKD, inflammation, and increased ROS	**-**	**+**	[1, 78, 79]
6	Sleep apnea	OSA upregulates VEGF and Ang-2 through nocturnal hypoxia, which activates HIF-1 and hypoxia-responsive RBPs	**-**	**+**	[40, 77]
7	Inflammation	ROS increases VEGF, Ang-2, TNF-α, and related mediators through activation of HIF-1, NF-κB, AP-1, and other transcriptional regulators	**+**	**+**	[52, 53, 66–71]

Abbreviations: +, positive effect (improve); -, negative effect (worsen); o, no effect; o+, improve indirectly; AGEs, advanced glycation endproducts; Ang-2, angiopoietin-2; ANG II, angiotensin II; CKD, chronic kidney disease; DR, diabetic retinopathy; HIF-1, hypoxia-inducible factor 1; HTN, hypertension; IL, interleukin; IR, insulin resistance; LDL, low-density lipoprotein; NF-κB, nuclear factor kappa-light-chain-enhancer of activated B cells; NVU, neurovascular unit; OSA, obstructive sleep apnea; ox-LDL, oxidized low-density lipoprotein; PI3K, phosphoinositide 3-kinase; PKC, protein kinase C; RBP, RNA-binding protein; ROS, reactive oxygen species; SBP, systolic blood pressure; SP1, specificity protein 1; TG, triglyceride; TNF-α, tumor necrosis factor-alpha; VEGF, vascular endothelial growth factor

Explained in text Section “Systemic and retinal effects of GLP-1”.

**Table 3:** Mixed reports on the DR risks of insulin and insulin secretagogues

	Clinical Observations	Explanations Based on the Angiogenesis-Inflammation Hypothesis
1	Early worsening of DR during first 2 years of insulin therapy	Due to insulin’s pro‑angiogenic properties and the hyperinsulinemia characteristic of insulin‑resistant states
2	Long-term DR risk reduction with insulin therapy 6 to 6.5 years	The long‑term benefits of sustained glycemic control and improved insulin sensitivity outweigh the transient early angiogenic surge induced by insulin.
3	Worsening of DR with SU therapy in short-term trials	SUs are insulin secretagogues that increase VEGF and Ang-2 through their insulinotropic action.
4	Lon-term DR risk reduction with SU therapy 6 to 10 years	Sustained glycemic control and improved insulin sensitivity ultimately outweigh the initial angiogenic surge triggered by the insulinotropic action of SUs.
5	Increased DR risk with DPP-4is during the first-year treatment, risk reduction with long‑term (≥10‑year) therapy	DPP-4is prevent the enzymatic degradation of endogenous GLP-1 and GIP , enhancing insulin secretion and potentially producing a biphasic DR effect like insulin.
6	Worsening of DR during the first 2 years of semaglutide therapy	Semaglutide is an insulin secretagogue that increases VEGF and Ang‑2 through its insulinotropic action.
7	Mixed reports on the DR risk of semaglutide	The treatment durations and comparators affect the results of a possible biphasic impact
8	Mixed reports on the DR risk of tirzepatide	Tirzepatide is an insulin secretagogue that increases VEGF and Ang‑2 through its insulinotropic action. The treatment durations and comparators affect the results of a possible biphasic impact

Abbreviations: Ang-2, angiopoietin-2; DPP-4i, dipeptidyl phosphatase-4 inhibitor; DR, diabetic retinopathy; GIP, glucose-dependent insulinotropic polypeptide; GLP-1, glucagon-like peptide-1; SU, sulfonylurea; VEGF, vascular endothelial growth factor.

Explained in text Section “Controversies in the retinopathy risk of semaglutide”.

## Potential clinical applications of the angiogenesis–inflammation hypothesis

The Angiogenesis–Inflammation Hypothesis does not account for all clinical findings in DR. For instance, the early thickening of the retinal capillary basement membrane is more plausibly attributed to non-enzymatic glycosylation, which leads to cross-linking of proteins and lipids within the membrane [90]. Nonetheless, many other features of DR are consistent with this hypothesis (Table 4). Chronic and poorly controlled diabetes is associated with increased production of angiogenic and inflammatory mediators, driven by hyperglycemia and the accumulation of AGEs [90]. Interconnected positive feedback loops involving hypoxia and inflammation may accelerate DR progression from stage 1 mild NPDR to stage 4 PDR (Fig. 2). The exacerbation of DR during puberty and pregnancy can be partially explained by the upregulation of VEGF and Ang-2, mediated by IGF-1 [14, 15, 27, 28], estrogen and progesterone [91, 92], in addition to hyperinsulinemia observed in both conditions. Postoperative hyperinsulinemia may account for the transient worsening of DR observed shortly after gastric bypass or pancreas transplantation, followed by long-term stabilization or improvement in retinopathy [9, 78, 93].

**Table 4:** Potential clinical applications of the angiogenesis-inflammation hypothesis

	Clinical Observations and Applications	Possible Explanations Based on the Angiogenesis-Inflammation Hypothesis
1	Worse DR with prolonged and poor glycemic control	Increased VEGF, Ang‑2, TNF‑α, and related mediators due to prolonged and severe hyperglycemia and greater AGEs accumulation
2	Acceleration of DR progression from stage 1 to stage 4	Positive feedback loops involving hypoxia and inflammation are activated.
3	Worsening DR during puberty	Increased VEGF and Ang-2 by IGF-1 and hyperinsulinemia in the insulin-resistant (IR) state
4	Worsening DR during pregnancy	Increased VEGF and Ang-2 by estrogen, progesterone, and hyperinsulinemia in IR state
5	Transient worsening of DR after gastric bypass and pancreas transplantation	Short-term post-operative hyperinsulinemia in IR state, and long-term improvement with sustained glycemic control and improved insulin sensitivity
6	Empagliflozin slows DR progression without early worsening of DR	Glycemic control by inhibiting renal glucose reabsorption independently of insulin; reduces retinal VEGF and Ang-2 and suppresses retinal inflammation
7	Combination therapy with empagliflozin and semaglutide for T2D	Mitigate semaglutide‑associated early DR risk by suppressing angiogenic and inflammatory mediators with empagliflozin
8	Mixed reports on the DR risks of SGLT-2i	Because insulin and SU comparators also reduce DR risk with sustained use, they may partially mask the distinct protective effects of SGLT‑2i.
9	Metformin slows DR progression without early worsening of DR	Insulin‑independent glycemic control by reducing hepatic glucose production; metformin also suppresses the production of angiogenic and inflammatory mediators in retina.
10	Combination therapy with Metformin and semaglutide for T2D	Mitigate the early DR risk of semaglutide by suppressing angiogenic and inflammatory mediators with metformin
11	All glucose lowering agents are expected to reduce DR risk after long-term treatment (> 6y)	Chronic hyperglycemia is the major risk factor for DR and the primary stimulus for the production of angiogenic and inflammatory mediators in retina
12	Long-term semaglutide or tirzepatide therapy is expected to reduce DR risk after 6y, like insulin and SU in DCCT and UKPDS	Sustained glycemic control and improved insulin sensitivity by semaglutide or tirzepatide outweigh the early angiogenic spike triggered by their insulinotropic action. They mitigate a broad spectrum of systemic DR risk factors and offer direct neurovascular protection in retina.
13	Predict a biphasic effect of tirzepatide on DR with early worsening followed by up to an 80% reduction in long-term risk	Both GIP and GLP-1 augment glucose-induced insulin secretion. Dual incretin-receptor activation by tirzepatide lowers HbA1c by up to 2.58%.
14	Mechanisms of PRP in treating PDR	Reduce VEGF, Ang-2, and inflammatory cytokine production from ischemic retinal areas, while also decreasing oxygen demand in the remaining viable retina
15	Mechanisms of vitrectomy in treating DME and PDR	Remove VEGF, Ang-2, TNF-α, and the scaffold for neovascularization from vitreous, and release VMT
16	Steroids effective for some DMEs refractory to anti-VEGF therapy	Decreases retinal VEGF, Ang-2 and inflammatory cytokine production
17	Limited efficacy of Pegaptanib (Macugen) in treating DME	Targets VEGF_165_ only, not the 3 other isoforms VEGF_121_, VEGF_189_ and VEGF_206_
18	Good efficacy of Bevacizumab and Ranibizumab for DME	Blocks all VEGF isoforms by Bevacizumab (Avastin) and Ranibizumab (Lucentis)
19	Excellent efficacy of Aflibercept 2 mg in treating DME	Targets both VEGF and PlGF
20	Superb efficacy of Aflibercept 8 mg in treating DME	4x higher dose of Aflibercept 8 mg (Eylea HD) than Aflibercept 2 mg (Eylea)
21	Superb efficacy of Faricimab (Vabysmo) in treating DME	Targeting both VEGF and Ang-2
22	Anti-VEGFs can slow DR progression and decrease DME	VEGF plays a central role in DR pathogenesis and DME formation
23	Semaglutide or tirzepatide add-on to insulin therapy for T1D with obesity	Semaglutide and tirzepatide mitigate a broader spectrum of DR risk factors than insulin, such as HTN, dyslipidemia, CKD and obesity, and may provide direct neurovascular protection to retina through GLP-1 receptor activation.
24	Triple therapy with metformin, an SGLT-2i and a GLP-1RA for T2D patients with high DR risk	Triple therapy targets glycemic control, weight loss, cardiovascular and renal protection, while maximally suppressing angiogenic and inflammatory mediators in the retina.

Abbreviations: AGEs, advanced glycation endproducts; Ang-2, angiopoietin-2; DCCT, Diabetes Control and Complications Trial; DME, diabetic macular edema; DR, diabetic retinopathy; GIP, glucose-dependent insulinotropic polypeptide; GLP-1, glucagon-like peptide-1; IGF-1, insulin-like growth factor-1; IR, insulin resistance; PDR, proliferative diabetic retinopathy; PlGF, placental growth factor; PRP, panretinal laser photocoagulation; SGLT-2i, sodium-glucose cotransporter 2 inhibitor; T1D, type 1 diabetes; T2D, type 2 diabetes; TNF-α, tumor necrosis factor-alpha; UKPDS, UK Prospective Diabetes Study; VEGF, vascular endothelial growth factor; VMT, vitreomacular traction; y, year

Explained in text Section “Potential clinical applications of the angiogenesis–inflammation hypothesis”

Both empagliflozin and metformin have been shown to slow DR progression without causing early worsening [6–8]. These agents reduce the production of angiogenic and inflammatory mediators in the retina not only through glycemic control but also by directly mitigating retinal inflammation [94–96]. Therefore, combination therapy with empagliflozin and metformin may offer additive glucose-lowering benefits without significant hypoglycemia risk, while potentially reducing the DR risk associated with semaglutide. Real-world data from a global federated database suggests that combining SGLT-2i with insulin therapy may reduce the incidence of DME [97]. Based on the UKPDS findings that insulin, SUs and metformin all reduced DR risk after six years of treatment, it is reasonable to infer that most glucose-lowering agents confer similar benefits with long-term use. Both semaglutide and tirzepatide are anticipated to exert a biphasic effect on DR like insulin, with tirzepatide potentially achieving up to an 80% reduction in long-term DR risk, corresponding to its reported 2.58% HbA1c decrease [64].

PRP treats PDR by reducing VEGF, Ang-2, and inflammatory cytokine production from ischemic retinal areas, while also decreasing oxygen demand in the remaining viable retina [19, 20]. Pegaptanib sodium (Macugen) has limited efficacy in the treatment of DME because it selectively inhibits only the VEGF_165_ isoform, without targeting the three other pathological isoforms—VEGF_121_, VEGF_189_, and VEGF_206_ [98]. In contrast, bevacizumab (Avastin) and ranibizumab (Lucentis) are effective in treating both DME and PDR by inhibiting all VEGF isoforms [23]. Aflibercept 2 mg (Eylea) demonstrates better efficacy than bevacizumab and ranibizumab in severe DME by targeting both VEGF and PlGF [23]. Faricimab (Vabysmo), which simultaneously inhibits Ang-2 and VEGF, has shown excellent efficacy and durability in DME treatment [19, 26]. Aflibercept 8 mg (Eylea HD), with a fourfold higher dose than the standard formulation, allows for extended treatment intervals [24]. Intravitreal corticosteroids are also effective in DME management by suppressing both angiogenesis and inflammation [10]. The ability of anti-VEGF agents to slow DR progression and reduce DME underscores the central role of VEGF in DR pathogenesis [25].

## Strategies to reduce DR risk of insulin and semaglutide

Vision loss from DR is largely preventable or reversible with close monitoring according to established guidelines and timely intervention at the onset of VTDR [12, 25]. Incorporating artificial intelligence (AI)-based screening may be added to optimize patient adherence to screening and treatment while reducing clinic workload [99]. Because DME can impair vision independently of DR stage, screening for macular edema is essential at every DR evaluation. Use of GLP-1RAs in T2D is not contraindicated by DR, but vigilant retinal monitoring is advised, particularly in patients with pre-existing DR.

In patients with T1D and obesity, combination therapy with insulin and semaglutide has been shown to improve HbA1c and reduce body weight without increasing the risk of hypoglycemia or diabetic ketoacidosis [82, 83]. Since high baseline HbA1c and the magnitude of its reduction are key risk factors for early DR worsening [2–4], gradual dose escalation of both insulin and semaglutide may reduce the risk of transient DR deterioration by moderating the pro-angiogenic effect of insulin. For patients with T2D and obesity, metformin and empagliflozin may be considered before or alongside insulin or semaglutide for their potential protective effect against DR [6–8, 87]. Gradual dose escalation of semaglutide within this triple regimen may reduce the risk of transient DR worsening by moderating its insulinotropic effect. A small risk of retinal vein occlusion has been associated with SGLT-2is [100], warranting further investigation to clarify this association and guide clinical decision-making. Table 5 summarizes strategies to mitigate vision loss risk when using insulin and semaglutide.

**Table 5:** Strategies to reduce the DR risk of insulin and semaglutide

	For both T1D and T2D	Rationale
1	Vigilant screening and close follow up	To assess baseline DR, especially the presence of VTDR
2	Prompt treatment for VTDR	Effective treatments are available with anti-VEGF therapy, laser photocoagulation, and vitrectomy to prevent vision loss
3	Add AI screening	To optimize patient adherence to screening and treatment while reducing clinic workload
	**For T1D with obesity**	
4	Combination therapy: Insulin + Semaglutide (or Tirzepatide) and slow dose escalation of both	Adding semaglutide or tirzepatide to insulin therapy lowers HbA1c and body weight without increasing the risk of hypoglycemia or diabetic ketoacidosis
	**For T2D with obesity**	
5	Metformin + EMPA before Semaglutide or Tirzepatide, and slow dose escalation of Semaglutide or Tirzepatide	To lower baseline HbA1c prior to initiating semaglutide or tirzepatide therapy and to temper their insulinotropic effects
6	Metformin + EMPA + Semaglutide or Tirzepatide, and slow dose escalation of Semaglutide or Tirzepatide	Metformin and empagliflozin are insulin‑independent anti‑T2D agents that may help mitigate the early DR worsening associated with semaglutide and tirzepatide.

Abbreviations: AI, artificial intelligence; DR, diabetic retinopathy; EMPA, empagliflozin; HbA1c, hemoglobin A1c; T1D, type 1 diabetes; T2D, type 2 diabetes; VTDR, vision threatening diabetic retinopathy

Explained in text Section “Strategies to reduce DR risk of insulin and semaglutide”.

## Strengths and limitations

This article proposes a novel hypothesis for the pathogenesis of DR by integrating established biochemical, hemodynamic, and endocrine theories with the roles of angiogenic growth factors and inflammatory cytokines, particularly VEGF and Ang-2. The supporting evidence is reviewed, emphasizing the interplay between angiogenesis and inflammation via endothelial cell adhesion molecules, and highlighting the dual pro-angiogenic and pro-inflammatory properties of VEGF and Ang-2. This integrated hypothesis offers a comprehensive framework that accounts for many clinical observations in DR, including the paradoxical worsening of DR associated with insulin and semaglutide therapies. It suggests that concurrently targeting both angiogenic growth factors and inflammatory cytokines may yield optimal therapeutic efficacy in the treatment of DR, especially DME [14]. This review establishes a compelling link between molecular mechanisms and clinical observations and predicts that long-term use of GLP-1-based therapies may reduce DR risk by improving glycemic control, addressing additional risk factors more effectively than insulin alone, and providing direct retinal neuroprotection through GLP-1 receptor activation. It underscores the importance of trial duration and choice of comparator in interpreting published findings and designing future studies, as both factors can significantly influence outcomes, potentially leading to opposite conclusions. 

Importantly, the Angiogenesis–Inflammation Hypothesis challenges the long‑standing assumption that rapid glycemic improvement or large reductions in HbA1c are responsible for the early worsening of DR observed with insulin and semaglutide therapies—a view that is difficult to reconcile with robust clinical trial evidence establishing hyperglycemia itself as the primary driver of DR [4, 48, 54]. Empagliflozin lowers glucose rapidly and produces early HbA1c reductions [88] yet has been shown to be protective against DR [6], whereas semaglutide—despite requiring slow dose titration and achieving a more gradual decline in HbA1c [1, 88]—has been associated with early worsening [2, 3]. In terms of magnitude, a similar inconsistency is observed with DPP‑4is, which produce substantially smaller HbA1c reductions than metformin [88] yet have been associated with early DR deterioration [58], whereas metformin—despite achieving larger HbA1c reductions—has shown no such signal [7, 8, 87]. Moreover, when early worsening occurred with intensive insulin therapy, it was transient, non‑progressive, and the magnitude of HbA1c reduction did not predict long‑term harm [4]. In fact, large improvements in HbA1c conferred substantial long‑term retinopathy protection in both the DCCT and UKPDS trials [4, 48, 54]. Collectively, these discordant patterns indicate that neither the speed nor the magnitude of glycemic improvement adequately explains early worsening of DR, pointing instead toward drug‑specific or pathway‑mediated mechanisms rather than a purely glycemic one. This review suggests that early worsening of DR appears to be insulin‑specific—arising either from exogenous insulin therapy or from endogenous insulin secretion stimulated by secretagogues such as SUs, DPP‑4is, and GLP‑1RAs [2–5, 9, 49, 58]—and does not occur with insulin‑independent agents such as metformin or empagliflozin [6–8]. It summarizes evidence supporting insulin’s pro‑angiogenic effects and its pro‑inflammatory actions in insulin‑resistant states. A key therapeutic implication of this new hypothesis is that combining semaglutide with both empagliflozin and metformin (triple therapy) may mitigate semaglutide‑associated early DR worsening. Triple therapy addresses multiple facets of type 2 diabetes—glycemic control, weight reduction, and cardiovascular and renal protection—and, as illustrated in Figure 7, the combined protective effects of metformin and empagliflozin are predicted to counterbalance the transient DR‑exacerbating influence of semaglutide. Although prospective trials are needed to validate the retinal benefits of triple therapy, current guidelines support early dual therapy for T2D with additional agents added individually based on patient-specific factors [101], making it an appealing strategy for individuals at elevated risk of DR progression.

While this review infers that semaglutide’s DR risk may stem from insulin’s pro-angiogenic actions, direct evidence is currently limited. Future preclinical and clinical studies are needed to elucidate whether GLP-1RAs directly induce angiogenic and inflammatory responses in retinal tissue. Although preclinical studies demonstrate insulin’s pro-angiogenic potential, whether comparable concentrations of insulin can be achieved locally in the retina through exogenous insulin therapy remains uncertain. Insulin may influence retinal pathology indirectly via systemic metabolic changes that contribute to early worsening of DR. Additionally, it is unclear whether the observed DR risk is unique to semaglutide or represents a class effect of GLP-1-based therapies. Although a retrospective cohort study reported that tirzepatide accelerated DR progression from moderate or severe NPDR to PDR [102], robust evidence from randomized trials is still lacking, underscoring the need for more RCTs on the DR risk of tirzepatide. While most existing studies focus on the progression of DR, there remains a research gap regarding the effects of GLP-1RAs on the onset of DR and the development of DME.

## Conclusions

The early worsening of DR observed with insulin therapy may be more accurately attributed to insulin’s pro-angiogenic effects rather than solely to the rapid glycemic correction. As a glucose-dependent insulin secretagogue, semaglutide may likewise cause early DR progression. Long-term semaglutide therapy, however, is anticipated to reduce DR risk by significantly improving glycemic control and addressing a broader spectrum of metabolic risk factors - such as HTN, dyslipidemia, CKD, obesity, and OSA - more effectively than insulin, while also providing direct neurovascular protection through retinal GLP-1 receptor activation. In patients with T1D, adding semaglutide to insulin therapy may provide additional long‑term protection against retinopathy. In patients with T2D, combination therapy that includes insulin‑independent agents such as metformin and empagliflozin may help mitigate the transient increase in DR risk associated with insulin, semaglutide, and other insulin secretagogues.

## Supplementary Information

Below is the link to the electronic supplementary material.

Supplementary Material 1

Supplementary Material 2

Supplementary Material 3

## Data Availability

No datasets were generated or analysed during the current study.
