# Supplementary figures and images for: Angiogenesis and inflammation in the retinopathy risk of insulin and semaglutide – a review
Source: Int J Retina Vitreous. 2026 Mar 18;12:67. doi: 10.1186/s40942-026-00811-8 (PMC13113116; doi:10.1186/s40942-026-00811-8)

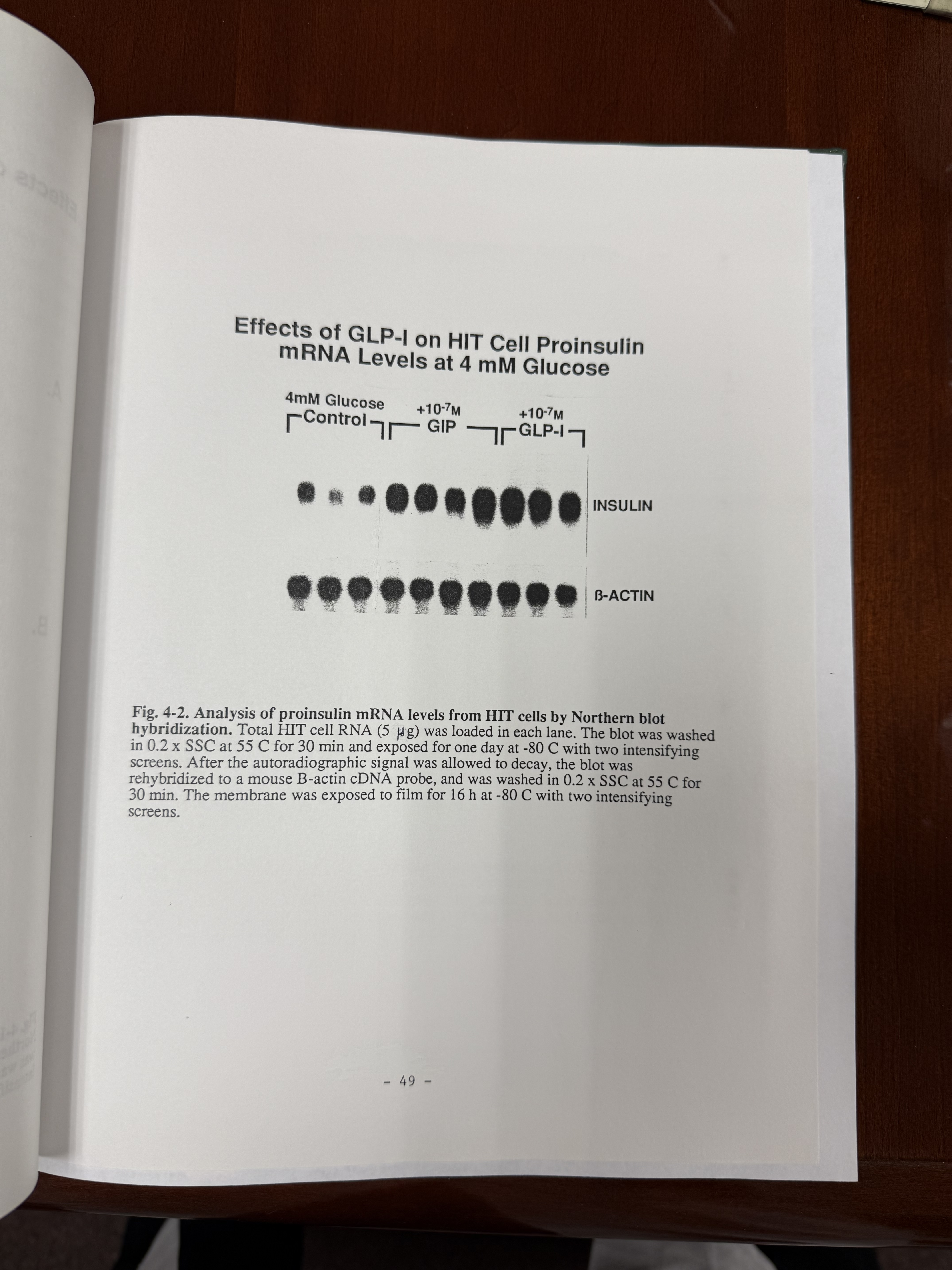

Supplement: Supplementary file 1 — Supplementary Material 1 [file 40942_2026_811_MOESM1_ESM.tiff]
