# Supplementary material for: Angiogenesis and inflammation in the retinopathy risk of insulin and semaglutide – a review
Source: Int J Retina Vitreous. 2026 Mar 18;12:67. doi: 10.1186/s40942-026-00811-8 (PMC13113116; doi:10.1186/s40942-026-00811-8)
Supplement: Supplementary file 2 — Supplementary Material 2 [file 40942_2026_811_MOESM2_ESM.pdf]

mAb (1) was added in some wells, and a previously characterized anti-gp120 mAb (1) was added to other wells as a control. After 48 h, 10 μl (0.2 μCi) of [^{3}H]thymidine (specific activity 27 mCi/mg) was added for 6 h, and [^{3}H]thymidine incorporation into DNA was determined by liquid scintillation counting. Values were normalized to cell number.

Statistics. Significance testing was done using the paired Student's *t* test. P values < 0.05 were deemed significant.

Results

To examine whether AGEs increase retinal VEGF mRNA levels in vivo, AGEs were injected into the vitreous of rat and rabbit eyes, and in situ hybridization studies and Northern blot analyses were completed. 4 h after the injection of nonglycated BSA or AGE-BSA (final vitreous concentration 100 μg/ml), rat retinal VEGF mRNA levels were increased in the ganglion, inner nuclear, proximal photoreceptor, RPE, and choroidal layers of the AGE-injected rat eyes (Fig. 1, *A–C*). Northern blot analyses of rabbit neurosensory retina identified a 4.8-fold increase in VEGF mRNA levels in the AGE-injected eyes (Fig. 1 *D*).

Human RPE and bovine smooth muscle cell lines were used to define the mechanisms by which AGEs stimulate VEGF gene expression. AGEs (100 μg/ml) increased RPE VEGF mRNA to peak levels within 4 h, and the increases were sustained for at least 20 h (data not shown). VEGF mRNA levels were increased by as little as 1 μg/ml AGE, peaked with 100 μg/ml, and had an ED_{50} of 35 μg/ml. AGE levels have been reported to be between 25–80 μg/ml in the serum of human diabetic patients (12). A recent report demonstrated similar in vitro AGE-induced VEGF increases, which could be blocked with an anti-AGE antibody (25).

Up to four VEGF isoforms are produced by a single gene (26, 27). RNase protection assays showed that VEGF121 increased 4.9±0.4-fold after exposure to 100 μg/ml AGEs for 8 h and represented 54% of the VEGF mRNA pool (Fig. 2 *A*, n = 4, $P < 0.01$). The band representing VEGF165, 189, and 206 was increased to a similar degree (4.2±0.3-fold, n = 4, $P < 0.01$). Since ischemic hypoxia is a pathophysiologically relevant stimulus for retinal VEGF gene expression (1), the effect of AGEs and hypoxia on VEGF gene expression was examined. Human RPE cells were exposed to hypoxia (3% O_2) or normoxia (21% O_2) for 6 h, with or without 100 μg/ml AGEs. Hypoxia and AGEs alone increased VEGF mRNA 4.5- and 5.0-fold, respectively. The combination of hypoxia and AGEs increased VEGF mRNA levels 10.8-fold (Fig. 2 *B*). Since it has been shown that AGE-induced increases in gene expression can occur through ROI (13), the ability of AGEs to stimulate VEGF gene expression by similar mechanisms was examined. The antioxidants DMTU and NAC blocked completely the AGE-induced increases in VEGF mRNA in bovine SMCs and RPE cells (Fig. 2, *C* and *D*), implicating ROI as mediators of the VEGF response.

Finally, the AGE-associated increases in VEGF mRNA levels were translated into secreted bioactive VEGF protein. After 24 h of incubation, AGEs (100 μg/ml) increased VEGF protein levels in the conditioned media of RPE cells by 1.7±0.1-fold (n = 6, $P < 0.001$) (Fig. 3 *A*). The increases occurred in a time-dependent manner from 8 to 24 h (data not shown). The bioactivity of the secreted VEGF protein was examined with BCE DNA synthesis assays. Conditioned media from AGE-treated RPE cells increased significantly capillary endothelial DNA synthesis 2.0±0.2-fold (n = 3, $P < 0.01$) (Fig. 3 *B*). The identity of the endothelial cell mitogen in the conditioned media was examined using a previously characterized

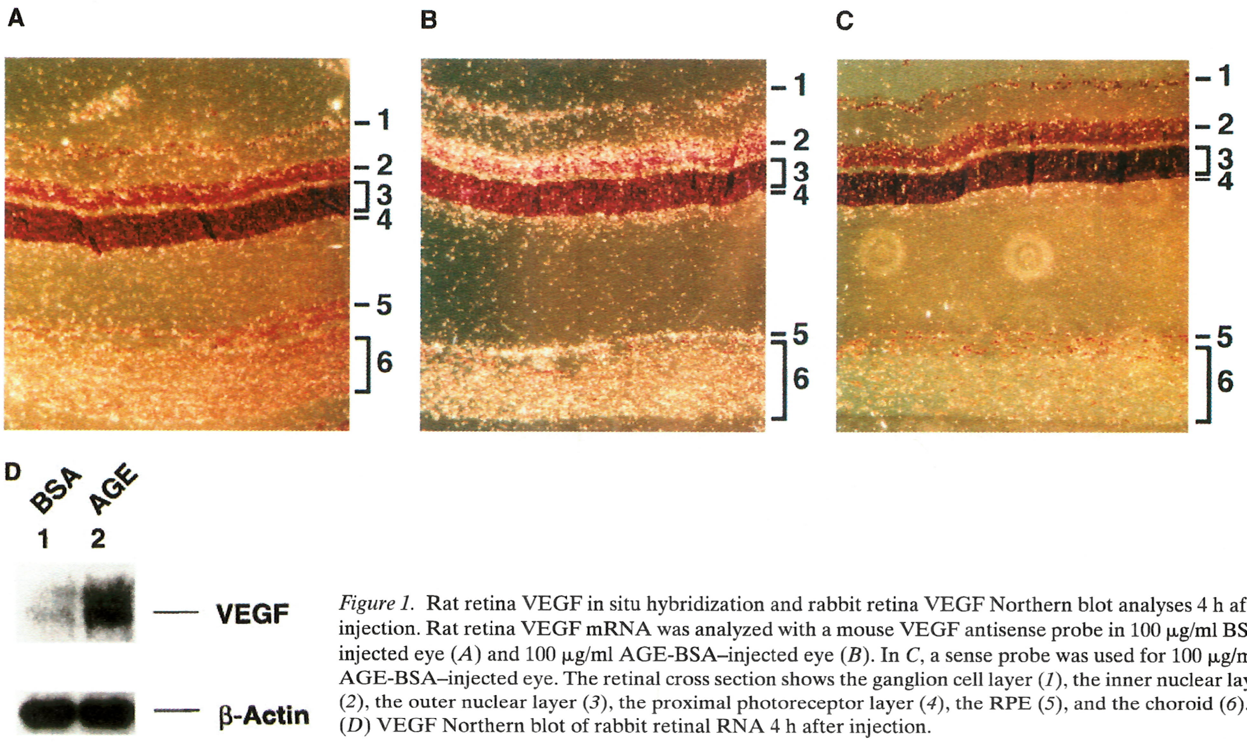

Figure 1. Rat retina VEGF in situ hybridization and rabbit retina VEGF Northern blot analyses 4 h after injection. Rat retina VEGF mRNA was analyzed with a mouse VEGF antisense probe in 100 μg/ml BSA–injected eye (*A*) and 100 μg/ml AGE-BSA–injected eye (*B*). In *C*, a sense probe was used for 100 μg/ml AGE-BSA–injected eye. The retinal cross section shows the ganglion cell layer (*1*), the inner nuclear layer (*2*), the outer nuclear layer (*3*), the proximal photoreceptor layer (*4*), the RPE (*5*), and the choroid (*6*). (*D*) VEGF Northern blot of rabbit retinal RNA 4 h after injection.

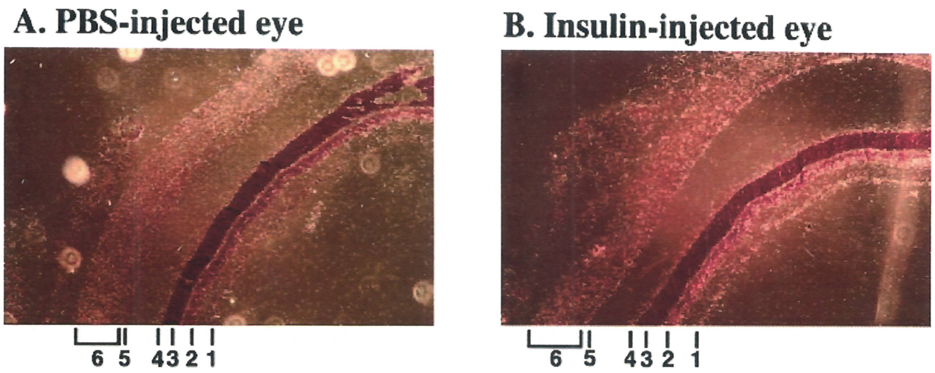

Figure 1. VEGF in situ hybridization of rat retina 2 hours after injection of insulin or vehicle solution. Rat retina VEGF mRNA levels were analyzed with a mouse VEGF antisense probe in PBS-injected (**A**) and insulin-injected (**B**) eyes. The final concentration of insulin in the rat vitreous was 50 nM, based on a volume for the rat vitreous of 100 μl. The retinal cross-section shows the ganglion cell layer (1), the inner nuclear layer (2), the outer nuclear layer (3), the proximal photoreceptor layer (4), the RPE (5), and the choroid (6).

control anti-gp120[22] antibody was added to some wells for the neutralization study groups.

The cells were washed with PBS, fixed with 100% ethanol for 5 minutes, washed with borate buffer (0.1 M; pH 8.5), and stained with methylene blue (1% in borate buffer) for 10 minutes and rinsed with tap water. After 30 minutes of color extraction with 0.1 N HCl, the cell density was quantified with an ELISA reader at 600 nm.[35]

Statistics

Significance testing was performed using the paired Student's *t*-test. $P < 0.05$ was deemed significant.

Results

To examine whether insulin increases VEGF mRNA in vivo, 10 μl insulin (final concentration 50 nM) was injected into the vitreous of rats. We used 50 nM for this study because this concentration of insulin gave a maximal response (see Figs. 3, 5). Using in situ hybridization, VEGF mRNA levels were seen to be elevated in the insulin-injected eyes compared with contralateral eyes receiving 10 μl vehicle alone (PBS). VEGF mRNA levels were increased in the ganglion, inner nuclear, and RPE cell layers (Fig. 1). The insulin-induced increases in VEGF mRNA levels were characterized in human RPE cells in vitro. The RNase protection assay was performed using a riboprobe corresponding to $VEGF_{121}$ (416-nt protected fragment). This probe also detects three other isoforms of VEGF as a single protected fragment (338 nt).[37] At 2 hours there was an increase of 5.2 ± 0.4-fold ($n = 3$) in the level of $VEGF_{121}$ mRNA in 50 nM insulin-treated RPE cells (Fig. 2). The band corresponding to $VEGF_{165}$, $VEGF_{189}$, and $VEGF_{206}$ was also significantly increased (4.2 ± 0.3-fold; $n = 3$).

The ability of insulin to stimulate the release of VEGF protein into the conditioned media of RPE cells was tested. Human RPE cells exposed to insulin for 24 hours increased VEGF protein levels in conditioned media in a dose-dependent manner, with a median effective dose (EC_{50}) of 5 nM (Fig. 3). The bioactivity of the RPE-conditioned media was determined using BCE cell proliferation assays. Conditioned media from 50 nM insulin-treated RPE cells increased BCE cell density 1.65 ± 0.23-fold, compared with the untreated RPE cell media ($n = 3$; $P < 0.05$; Fig. 4), an effect that was completely blocked by the anti-VEGF neutralizing antibody, but not the isotype control anti-gp120 antibody (Fig. 4).

To examine whether the increase in VEGF mRNA levels by insulin was due to an enhancement of VEGF transcription, the effect of insulin on VEGF promoter activity was examined in transient transfection assays. Insulin (5–50 nM) stimulated VEGF promoter activity in a dose-dependent manner (EC_{50}, 9 nM; $n = 3$), with the maximal effect at 50 nM insulin (2.8 ± 0.2-fold; $n = 3$; $P < 0.01$). In the control, 50 nM insulin did not affect the activity of the 81-bp thymidine kinase promoter (1.0 ± 0.1-fold; $n = 3$; Fig. 5).

The RNase protection assay showed that the addition of actinomycin D to RPE cells 1 hour before insulin treatment abrogated the insulin-induced increases in VEGF expression (Fig. 6A), suggesting that insulin increases VEGF expression mainly through enhanced transcription. To examine whether insulin also alters VEGF transcript stability, the RNase protection assay was performed to measure the half-life of VEGF transcripts in RPE cells after insulin treatment. The addition of 50 nM insulin did not significantly enhance the $VEGF_{121}$ transcript half-life in RPE cells (1.10 ± 0.13 hours versus 0.98 ± 0.09 hours; $n = 3$; $P > 0.05$; Fig. 6B).

Discussion

In this study, we found that insulin increased VEGF mRNA levels in the ganglion, inner nuclear, and RPE cell layers of the rat retina. In vitro, insulin increased VEGF mRNA and secreted protein levels in human RPE cells. The conditioned media of insulin-treated retinal cells stimulated capillary endothelial cell proliferation, a response that was blocked with an anti-VEGF

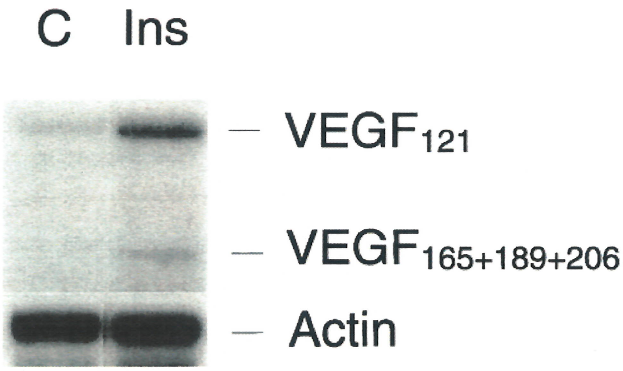

Figure 2. VEGF isoforms were coordinately increased by insulin. RNase protection assay 2 hours after insulin treatment of RPE cells showed protected fragments of expected size. The human β-actin mRNA was used to normalize the samples for quantification of isoform expression. C, control; Ins, 50 nM insulin.

Downloaded from iovs.arvojournals.org on 11/01/2023
