# Supplementary material for: Angiogenesis and inflammation in the retinopathy risk of insulin and semaglutide – a review
Source: Int J Retina Vitreous. 2026 Mar 18;12:67. doi: 10.1186/s40942-026-00811-8 (PMC13113116; doi:10.1186/s40942-026-00811-8)
Supplement: Supplementary file 3 — Supplementary Material 3 [file 40942_2026_811_MOESM3_ESM.pdf]

JOHN WILEY AND SONS LICENSE
TERMS AND CONDITIONS

Jan 16, 2026

This Agreement between ming lu ("You") and John Wiley and Sons ("John Wiley and Sons") consists of your license details and the terms and conditions provided by John Wiley and Sons and Copyright Clearance Center.

All payments must be made in full to CCC. For payment instructions, please see information listed at the bottom of this form.

License Number	6190620546823
License date	Jan 16, 2026
Licensed Content Publisher	John Wiley and Sons
Licensed Content Publication	Diabetes, Obesity and Metabolism
Licensed Content Title	Semaglutide, reduction in glycated haemoglobin and the risk of diabetic retinopathy
Licensed Content Author	Tina Vilsbøll, Stephen C. Bain, Lawrence A. Leiter, et al
Licensed Content Date	Jan 8, 2018
Licensed Content Volume	20
Licensed Content Issue	4
Licensed Content Pages	9
Type of use	Journal/Magazine

Requestor type	Publisher, not-for-profit
Is the reuse sponsored by or associated with a pharmaceutical or medical products company?	no
Format	Electronic
Portion	Figure/table
Number of figures/tables	1
Will you be translating?	No
Circulation	100 - 199
Title of new article	Angiogenesis and Inflammation in the Retinopathy Risk of Insulin and Semaglutide
Lead author	ming lu
Title of targeted journal	International Journal of Retina and Vitreous
Publisher	Springer Nature
Publisher imprint	BMC
Expected publication date	Jan 2026
Portions	Figure 1C
The Requesting Person / Organization to Appear on the License	ming lu
Requestor Location	Dr. Ming Lu<br>1327 Troup Highway<br>Eye Center<br><br>Tyler, TX 75701<br>United States

Order reference number	3
Publisher Tax ID	EU826007151
Payment Type	Credit Card
Credit card info	Visa ending in 2566
Credit card expiration	10/2030
Email Address	Ming.lu@christushealth.org
Billing Address	Ming Lu<br>1327 Troup Highway<br>Eye Center<br><br>Tyler, TX 75701<br>United States
Total	75.00 USD

Terms and Conditions

TERMS AND CONDITIONS

This copyrighted material is owned by or exclusively licensed to John Wiley & Sons, Inc. or one of its group companies (each a"Wiley Company") or handled on behalf of a society with which a Wiley Company has exclusive publishing rights in relation to a particular work (collectively "WILEY"). By clicking "accept" in connection with completing this licensing transaction, you agree that the following terms and conditions apply to this transaction (along with the billing and payment terms and conditions established by the Copyright Clearance Center Inc., ("CCC's Billing and Payment terms and conditions"), at the time that you opened your RightsLink account (these are available at any time at http://myaccount.copyright.com).

Terms and Conditions

- The materials you have requested permission to reproduce or reuse (the "Wiley Materials") are protected by copyright.

- You are hereby granted a personal, non-exclusive, non-sub licensable (on a stand-alone basis), non-transferable, worldwide, limited license to reproduce the Wiley Materials for the purpose specified in the licensing process. This license, **and any CONTENT (PDF or image file) purchased as part of your order,** is for a one-time use only and limited to any maximum distribution number specified in the license. The first instance of republication or reuse granted by this license must be completed within two years of the date of the grant of this license (although copies prepared before the end date may be distributed thereafter). The Wiley Materials shall not be used in any other manner or for any other purpose, beyond what is granted in the license. Permission is granted subject to an appropriate acknowledgement given to the author, title of the material/book/journal and the publisher. You shall also duplicate the copyright notice that appears in the Wiley publication in your use of the Wiley Material. Permission is also granted on the understanding that nowhere in the text is a previously published source acknowledged for all or part of this Wiley Material. Any third party content is expressly excluded from this permission.
- With respect to the Wiley Materials, all rights are reserved. Except as expressly granted by the terms of the license, no part of the Wiley Materials may be copied, modified, adapted (except for minor reformatting required by the new Publication), translated, reproduced, transferred or distributed, in any form or by any means, and no derivative works may be made based on the Wiley Materials without the prior permission of the respective copyright owner.**For STM Signatory Publishers clearing permission under the terms of the STM Permissions Guidelines only, the terms of the license are extended to include subsequent editions and for editions in other languages, provided such editions are for the work as a whole in situ and does not involve the separate exploitation of the permitted figures or extracts,** You may not alter, remove or suppress in any manner any copyright, trademark or other notices displayed by the Wiley Materials. You may not license, rent, sell, loan, lease, pledge, offer as security, transfer or assign the Wiley Materials on a stand-alone basis, or any of the rights granted to you hereunder to any other person.
- The Wiley Materials and all of the intellectual property rights therein shall at all times remain the exclusive property of John Wiley & Sons Inc, the Wiley Companies, or their respective licensors, and your interest therein is

only that of having possession of and the right to reproduce the Wiley Materials pursuant to Section 2 herein during the continuance of this Agreement. You agree that you own no right, title or interest in or to the Wiley Materials or any of the intellectual property rights therein. You shall have no rights hereunder other than the license as provided for above in Section 2. No right, license or interest to any trademark, trade name, service mark or other branding ("Marks") of WILEY or its licensors is granted hereunder, and you agree that you shall not assert any such right, license or interest with respect thereto

- NEITHER WILEY NOR ITS LICENSORS MAKES ANY WARRANTY OR REPRESENTATION OF ANY KIND TO YOU OR ANY THIRD PARTY, EXPRESS, IMPLIED OR STATUTORY, WITH RESPECT TO THE MATERIALS OR THE ACCURACY OF ANY INFORMATION CONTAINED IN THE MATERIALS, INCLUDING, WITHOUT LIMITATION, ANY IMPLIED WARRANTY OF MERCHANTABILITY, ACCURACY, SATISFACTORY QUALITY, FITNESS FOR A PARTICULAR PURPOSE, USABILITY, INTEGRATION OR NON-INFRINGEMENT AND ALL SUCH WARRANTIES ARE HEREBY EXCLUDED BY WILEY AND ITS LICENSORS AND WAIVED BY YOU.
- WILEY shall have the right to terminate this Agreement immediately upon breach of this Agreement by you.
- You shall indemnify, defend and hold harmless WILEY, its Licensors and their respective directors, officers, agents and employees, from and against any actual or threatened claims, demands, causes of action or proceedings arising from any breach of this Agreement by you.
- IN NO EVENT SHALL WILEY OR ITS LICENSORS BE LIABLE TO YOU OR ANY OTHER PARTY OR ANY OTHER PERSON OR ENTITY FOR ANY SPECIAL, CONSEQUENTIAL, INCIDENTAL, INDIRECT, EXEMPLARY OR PUNITIVE DAMAGES, HOWEVER CAUSED, ARISING OUT OF OR IN CONNECTION WITH THE DOWNLOADING, PROVISIONING, VIEWING OR USE OF THE MATERIALS REGARDLESS OF THE FORM OF ACTION, WHETHER FOR BREACH OF CONTRACT, BREACH OF WARRANTY, TORT, NEGLIGENCE, INFRINGEMENT OR OTHERWISE (INCLUDING, WITHOUT LIMITATION, DAMAGES BASED ON LOSS OF PROFITS, DATA, FILES, USE, BUSINESS OPPORTUNITY OR CLAIMS OF THIRD PARTIES), AND WHETHER OR NOT THE PARTY HAS BEEN ADVISED OF THE POSSIBILITY OF SUCH DAMAGES.

THIS LIMITATION SHALL APPLY NOTWITHSTANDING ANY FAILURE OF ESSENTIAL PURPOSE OF ANY LIMITED REMEDY PROVIDED HEREIN.

- Should any provision of this Agreement be held by a court of competent jurisdiction to be illegal, invalid, or unenforceable, that provision shall be deemed amended to achieve as nearly as possible the same economic effect as the original provision, and the legality, validity and enforceability of the remaining provisions of this Agreement shall not be affected or impaired thereby.
- The failure of either party to enforce any term or condition of this Agreement shall not constitute a waiver of either party's right to enforce each and every term and condition of this Agreement. No breach under this agreement shall be deemed waived or excused by either party unless such waiver or consent is in writing signed by the party granting such waiver or consent. The waiver by or consent of a party to a breach of any provision of this Agreement shall not operate or be construed as a waiver of or consent to any other or subsequent breach by such other party.
- This Agreement may not be assigned (including by operation of law or otherwise) by you without WILEY's prior written consent.
- Any fee required for this permission shall be non-refundable after thirty (30) days from receipt by the CCC.
- These terms and conditions together with CCC's Billing and Payment terms and conditions (which are incorporated herein) form the entire agreement between you and WILEY concerning this licensing transaction and (in the absence of fraud) supersedes all prior agreements and representations of the parties, oral or written. This Agreement may not be amended except in writing signed by both parties. This Agreement shall be binding upon and inure to the benefit of the parties' successors, legal representatives, and authorized assigns.
- In the event of any conflict between your obligations established by these terms and conditions and those established by CCC's Billing and Payment terms and conditions, these terms and conditions shall prevail.
- WILEY expressly reserves all rights not specifically granted in the combination of (i) the license details provided by you and accepted in the

course of this licensing transaction, (ii) these terms and conditions and (iii) CCC's Billing and Payment terms and conditions.

- This Agreement will be void if the Type of Use, Format, Circulation, or Requestor Type was misrepresented during the licensing process.
- This Agreement shall be governed by and construed in accordance with the laws of the State of New York, USA, without regards to such state's conflict of law rules. Any legal action, suit or proceeding arising out of or relating to these Terms and Conditions or the breach thereof shall be instituted in a court of competent jurisdiction in New York County in the State of New York in the United States of America and each party hereby consents and submits to the personal jurisdiction of such court, waives any objection to venue in such court and consents to service of process by registered or certified mail, return receipt requested, at the last known address of such party.

WILEY OPEN ACCESS TERMS AND CONDITIONS

Wiley Publishes Open Access Articles in fully Open Access Journals and in Subscription journals offering Online Open. Although most of the fully Open Access journals publish open access articles under the terms of the Creative Commons Attribution (CC BY) License only, the subscription journals and a few of the Open Access Journals offer a choice of Creative Commons Licenses. The license type is clearly identified on the article.

The Creative Commons Attribution License

The Creative Commons Attribution License (CC-BY) allows users to copy, distribute and transmit an article, adapt the article and make commercial use of the article. The CC-BY license permits commercial and non-

Creative Commons Attribution Non-Commercial License

The Creative Commons Attribution Non-Commercial (CC-BY-NC)License permits use, distribution and reproduction in any medium, provided the original work is properly cited and is not used for commercial purposes.(see below)

Creative Commons Attribution-Non-Commercial-NoDerivs License

The Creative Commons Attribution Non-Commercial-NoDerivs License (CC-BY-NC-ND) permits use, distribution and reproduction in any medium, provided the

original work is properly cited, is not used for commercial purposes and no modifications or adaptations are made. (see below)

Use by commercial "for-profit" organizations

Use of Wiley Open Access articles for commercial, promotional, or marketing purposes requires further explicit permission from Wiley and will be subject to a fee.

Further details can be found on Wiley Online Library http://olabout.wiley.com/WileyCDA/Section/id-410895.html

Other Terms and Conditions:

v1.10 Last updated September 2015

Questions? customercare@copyright.com.

ELSEVIER LICENSE TERMS AND CONDITIONS Jan 16, 2026

This Agreement between Ming Lu ("You") and Elsevier ("Elsevier") consists of your license details and the terms and conditions provided by Elsevier and Copyright Clearance Center. License Number 6190640244899

License date Jan 16, 2026

Licensed Content Publisher Elsevier

Licensed Content Publication The Lancet

Licensed Content Title

Intensive blood-glucose control with sulphonylureas or insulin compared with conventional treatment and risk of complications in patients with type 2 diabetes (UKPDS 33)

PDF form attached

Diabetic Retinopathy and Other Ocular Findings in the Diabetes Control and Complications Trial/Epidemiology of Diabetes Interventions and Complications Study

Author:

Aiello, Lloyd Paul; for the DCCT/EDIC Research Group

Publication:

Diabetes Care

Publisher:

John Wiley and Sons

Date:

Dec 11, 2013

Copyright © 2013, John Wiley and Sons

Open Access Article

This is an open access article distributed under the terms of the Creative Commons CC BY license, which permits unrestricted use, distribution, and reproduction in any medium, provided the original work is properly cited.

You are not required to obtain permission to reuse this article.

For an understanding of what is meant by the terms of the Creative Commons License, please refer to Wiley's Open Access Terms and Conditions.

Permission is not required for this type of reuse.

Wiley offers a professional reprint service for high quality reproduction of articles from over 1400 scientific and medical journals. Wiley's reprint service offers:

- Peer reviewed research or reviews
- Tailored collections of articles

- A professional high quality finish
- Glossy journal style color covers
- Company or brand customisation
- Language translations
- Prompt turnaround times and delivery directly to your office, warehouse or congress.

Please contact our Reprints department for a quotation.
Email corporatesaleseurope@wiley.com or corporatesalesusa@wiley.com or corporatesalesDE@wiley.com.

CCC Marketplace

This is a License Agreement between ming lu ("User") and Copyright Clearance Center, Inc. ("CCC") on behalf of the Rightsholder identified in the order details below. The license consists of the order details, the Marketplace Permissions General Terms and Conditions below, and any Rightsholder Terms and Conditions which are included below.

All payments must be made in full to CCC in accordance with the Marketplace Permissions General Terms and Conditions below.

**Order Date**	16-Jan-2026	**Type of Use**	Republish in a journal/magazine
**Order License ID**	1691179-1	**Publisher**	American Society for Clinical Investigation
**ISSN**	0021-9738	**Portion**	Image/photo/illustration

LICENSED CONTENT

**Publication Title**	The Journal of Clinical Investigation	**Country**	United States of America
**Date**	01/01/1924	**Rightsholder**	American Society for Clinical Investigation
**Language**	English	**Publication Type**	Journal

REQUEST DETAILS

**Portion Type**	Image/photo/illustration	**Distribution**	Worldwide
**Number of Images / Photos / Illustrations**	1	**Translation**	Original language of publication
**Format (select all that apply)**	Electronic	**Copies for the Disabled?**	No
**Who Will Republish the Content?**	Author of requested content	**Minor Editing Privileges?**	No
**Duration of Use**	Life of current edition	**Incidental Promotional Use?**	No
**Lifetime Unit Quantity**	Up to 499	**Currency**	USD
**Rights Requested**	Main product		

NEW WORK DETAILS

**Title**	Angiogenesis and Inflammation in the Retinopathy Risk of Insulin and Semaglutide	**Publisher Imprint**	BMC
**Author**	ming lu	**Expected Publication Date**	2026-01-16
**Publication**	International Journal of Retina and Vitreous	**Expected Size of the New Work (number of pages)**	10
**Publisher**	Springer Nature	**Standard Identifier**	ISSN: 2056-9920

ADDITIONAL DETAILS

**Order Reference Number**	2

**The Requesting Person / Organization to Appear on the License**	ming lu

REQUESTED CONTENT DETAILS

**Title, Description or Numeric Reference of the Portion(s)**	Advanced glycation end products increase retinal vascular endothelial growth factor expression	**Title of the Article / Chapter the Portion Is From**	Advanced glycation end products increase retinal vascular endothelial growth factor expression
**Editor of Portion(s)**	1219-1224	**Author of Portion(s)**	Lu M
**Volume / Edition**	101	**Issue, if Republishing an Article From a Serial**	N/A
**Page or Page Range of Portion**	1219-1224	**Publication Date of Portion**	1923-12-31

Marketplace Permissions General Terms and Conditions

The following terms and conditions ("General Terms"), together with any applicable Publisher Terms and Conditions, govern User's use of Works pursuant to the Licenses granted by Copyright Clearance Center, Inc. ("CCC") on behalf of the applicable Rightsholders of such Works through CCC's applicable Marketplace transactional licensing services (each, a "Service").

1) **Definitions.** For purposes of these General Terms, the following definitions apply:

"License" is the licensed use the User obtains via the Marketplace platform in a particular licensing transaction, as set forth in the Order Confirmation.

"Order Confirmation" is the confirmation CCC provides to the User at the conclusion of each Marketplace transaction. "Order Confirmation Terms" are additional terms set forth on specific Order Confirmations not set forth in the General Terms that can include terms applicable to a particular CCC transactional licensing service and/or any Rightsholder-specific terms.

"Rightsholder(s)" are the holders of copyright rights in the Works for which a User obtains licenses via the Marketplace platform, which are displayed on specific Order Confirmations.

"Terms" means the terms and conditions set forth in these General Terms and any additional Order Confirmation Terms collectively.

"User" or "you" is the person or entity making the use granted under the relevant License. Where the person accepting the Terms on behalf of a User is a freelancer or other third party who the User authorized to accept the General Terms on the User's behalf, such person shall be deemed jointly a User for purposes of such Terms.

"Work(s)" are the copyright protected works described in relevant Order Confirmations.

2) **Description of Service.** CCC's Marketplace enables Users to obtain Licenses to use one or more Works in accordance with all relevant Terms. CCC grants Licenses as an agent on behalf of the copyright rightsholder identified in the relevant Order Confirmation.

3) **Applicability of Terms.** The Terms govern User's use of Works in connection with the relevant License. In the event of any conflict between General Terms and Order Confirmation Terms, the latter shall govern. User acknowledges that Rightsholders have complete discretion whether to grant any permission, and whether to place any limitations on any grant, and that CCC has no right to supersede or to modify any such discretionary act by a Rightsholder.

4) **Representations; Acceptance.** By using the Service, User represents and warrants that User has been duly authorized by the User to accept, and hereby does accept, all Terms.

CCC Marketplace

This is a License Agreement between ming lu ("User") and Copyright Clearance Center, Inc. ("CCC") on behalf of the Rightsholder identified in the order details below. The license consists of the order details, the Marketplace Permissions General Terms and Conditions below, and any Rightsholder Terms and Conditions which are included below.

All payments must be made in full to CCC in accordance with the Marketplace Permissions General Terms and Conditions below.

**Order Date**	15-Jan-2026	**Type of Use**	Republish in a journal/magazine
**Order License ID**	1691167-1	**Publisher**	ASSOCIATION FOR RESEARCH IN VISION AND OPHTHALMOLOGY
**ISSN**	1552-5783	**Portion**	Image/photo/illustration

LICENSED CONTENT

**Publication Title**	Investigative ophthalmology & visual science	**Country**	United States of America
**Author / Editor**	Association for Research in Vision and Ophthalmology.	**Rightsholder**	Association for Research in Vision & Ophthalmology (ARVO)
**Date**	01/01/1977	**Publication Type**	e-Journal
**Language**	English	**URL**	http://www.iovs.org

REQUEST DETAILS

**Portion Type**	Image/photo/illustration	**Distribution**	Worldwide
**Number of Images / Photos / Illustrations**	1	**Translation**	Original language of publication
**Format (select all that apply)**	Electronic	**Copies for the Disabled?**	No
**Who Will Republish the Content?**	Author of requested content	**Minor Editing Privileges?**	No
**Duration of Use**	Life of current edition	**Incidental Promotional Use?**	No
**Lifetime Unit Quantity**	Up to 499	**Currency**	USD
**Rights Requested**	Main product		

NEW WORK DETAILS

**Title**	Angiogenesis and Inflammation in the Retinopathy Risk of Insulin and Semaglutide	**Publisher Imprint**	BMC
**Author**	ming lu	**Expected Publication Date**	2026-03-01
**Publication**	International Journal of Retina and Vitreous	**Expected Size of the New Work (number of pages)**	10
**Publisher**	Springer Nature	**Standard Identifier**	ISSN: 2056-9920

ADDITIONAL DETAILS

**Order Reference Number**	1	**The Requesting Person / Organization to Appear on the License**	ming lu

REQUESTED CONTENT DETAILS

**Title, Description or Numeric Reference of the Portion(s)**	December 1999 Volume 40, Issue 13 ‹ Issue › Jump To... Methods Results Discussion Free Retinal Insulin-Induced Vascular Endothelial Growth Factor Expression in Retina	**Title of the Article / Chapter the Portion Is From**	Insulin-Induced Vascular Endothelial Growth Factor Expression in Retina
		**Author of Portion(s)**	Association for Research in Vision and Ophthalmology.
**Editor of Portion(s)**	N/A	**Issue, if Republishing an Article From a Serial**	N/A
**Volume / Edition**	Vol.40	**Publication Date of Portion**	2026-01-15
**Page or Page Range of Portion**	3281-3286		

RIGHTSHOLDER TERMS AND CONDITIONS

The ARVO journals--Investigative Ophthalmology & Visual Science, Journal of Vision, and Translational Vision Science and Technology--became open access starting with articles published in 2016. To reuse content published in 2016 or later, please check the Creative Commons license on the article to see if your reuse falls within the license's terms. If it doesn't, please contact the authors (who hold the copyright) directly for permission. Neither CCC nor ARVO has the authority to grant permission to reuse content published 2016-present.

Marketplace Permissions General Terms and Conditions

The following terms and conditions ("General Terms"), together with any applicable Publisher Terms and Conditions, govern User's use of Works pursuant to the Licenses granted by Copyright Clearance Center, Inc. ("CCC") on behalf of the applicable Rightsholders of such Works through CCC's applicable Marketplace transactional licensing services (each, a "Service").

1) **Definitions.** For purposes of these General Terms, the following definitions apply:

"License" is the licensed use the User obtains via the Marketplace platform in a particular licensing transaction, as set forth in the Order Confirmation.

"Order Confirmation" is the confirmation CCC provides to the User at the conclusion of each Marketplace transaction. "Order Confirmation Terms" are additional terms set forth on specific Order Confirmations not set forth in the General Terms that can include terms applicable to a particular CCC transactional licensing service and/or any Rightsholder-specific terms.

"Rightsholder(s)" are the holders of copyright rights in the Works for which a User obtains licenses via the Marketplace platform, which are displayed on specific Order Confirmations.

"Terms" means the terms and conditions set forth in these General Terms and any additional Order Confirmation Terms collectively.

"User" or "you" is the person or entity making the use granted under the relevant License. Where the person accepting the Terms on behalf of a User is a freelancer or other third party who the User authorized to accept the General Terms on the User's behalf, such person shall be deemed jointly a User for purposes of such Terms.

"Work(s)" are the copyright protected works described in relevant Order Confirmations.

CCC Marketplace

This is a License Agreement between ming lu ("User") and Copyright Clearance Center, Inc. ("CCC") on behalf of the Rightsholder identified in the order details below. The license consists of the order details, the Marketplace Permissions General Terms and Conditions below, and any Rightsholder Terms and Conditions which are included below.

All payments must be made in full to CCC in accordance with the Marketplace Permissions General Terms and Conditions below.

**Order Date**	18-Jan-2026	**Type of Use**	Republish in a journal/magazine
**Order License ID**	1691570-1	**Publisher**	ASSOCIATION FOR RESEARCH IN VISION AND OPHTHALMOLOGY
**ISSN**	1552-5783	**Portion**	Image/photo/illustration

LICENSED CONTENT

**Publication Title**	Investigative ophthalmology & visual science	**Country**	United States of America
**Author / Editor**	Association for Research in Vision and Ophthalmology.	**Rightsholder**	Association for Research in Vision & Ophthalmology (ARVO)
**Date**	01/01/1977	**Publication Type**	e-Journal
**Language**	English	**URL**	http://www.iovs.org

REQUEST DETAILS

**Portion Type**	Image/photo/illustration	**Distribution**	Worldwide
**Number of Images / Photos / Illustrations**	2	**Translation**	Original language of publication
**Format (select all that apply)**	Electronic	**Copies for the Disabled?**	No
**Who Will Republish the Content?**	Author of requested content	**Minor Editing Privileges?**	No
**Duration of Use**	Life of current edition	**Incidental Promotional Use?**	No
**Lifetime Unit Quantity**	Up to 499	**Currency**	USD
**Rights Requested**	Main product		

NEW WORK DETAILS

**Title**	Angiogenesis and inflammation in the retinopathy risk of insulin and semaglutide	**Publisher Imprint**	BMC
**Author**	ming lu	**Expected Publication Date**	2026-01-31
**Publication**	International Journal of Retina and Vitreous	**Expected Size of the New Work (number of pages)**	10
**Publisher**	Springer Nature	**Standard Identifier**	ISSN: 2056-9920

ADDITIONAL DETAILS

**Order Reference Number**	3	**The Requesting Person / Organization to Appear on the License**	ming lu

REQUESTED CONTENT DETAILS

**Title, Description or Numeric Reference of the Portion(s)**	VEGF increases retinal vascular ICAM-1 expression in vivo	**Title of the Article / Chapter the Portion Is From**	VEGF increases retinal vascular ICAM-1 expression in vivo
**Editor of Portion(s)**	N/A	**Author of Portion(s)**	Association for Research in Vision and Ophthalmology.
**Volume / Edition**	Vol.40		
**Page or Page Range of Portion**	1808-1812	**Issue, if Republishing an Article From a Serial**	N/A
		**Publication Date of Portion**	1976-12-31

RIGHTSHOLDER TERMS AND CONDITIONS

The ARVO journals--Investigative Ophthalmology & Visual Science, Journal of Vision, and Translational Vision Science and Technology--became open access starting with articles published in 2016. To reuse content published in 2016 or later, please check the Creative Commons license on the article to see if your reuse falls within the license's terms. If it doesn't, please contact the authors (who hold the copyright) directly for permission. Neither CCC nor ARVO has the authority to grant permission to reuse content published 2016-present.

Marketplace Permissions General Terms and Conditions

The following terms and conditions ("General Terms"), together with any applicable Publisher Terms and Conditions, govern User's use of Works pursuant to the Licenses granted by Copyright Clearance Center, Inc. ("CCC") on behalf of the applicable Rightsholders of such Works through CCC's applicable Marketplace transactional licensing services (each, a "Service").

1) **Definitions.** For purposes of these General Terms, the following definitions apply:

"License" is the licensed use the User obtains via the Marketplace platform in a particular licensing transaction, as set forth in the Order Confirmation.

"Order Confirmation" is the confirmation CCC provides to the User at the conclusion of each Marketplace transaction. "Order Confirmation Terms" are additional terms set forth on specific Order Confirmations not set forth in the General Terms that can include terms applicable to a particular CCC transactional licensing service and/or any Rightsholder-specific terms.

"Rightsholder(s)" are the holders of copyright rights in the Works for which a User obtains licenses via the Marketplace platform, which are displayed on specific Order Confirmations.

"Terms" means the terms and conditions set forth in these General Terms and any additional Order Confirmation Terms collectively.

"User" or "you" is the person or entity making the use granted under the relevant License. Where the person accepting the Terms on behalf of a User is a freelancer or other third party who the User authorized to accept the General Terms on the User's behalf, such person shall be deemed jointly a User for purposes of such Terms.

"Work(s)" are the copyright protected works described in relevant Order Confirmations.

2) **Description of Service.** CCC's Marketplace enables Users to obtain Licenses to use one or more Works in accordance with all relevant Terms. CCC grants Licenses as an agent on behalf of the copyright rightsholder identified in the relevant

From: Legal <aamc.autoresponse@aamc.org>
Date: January 17, 2026 at 10:56:44 PM CST
To: minglumd@gmail.com
Subject: Case # 03337218: Legal Form Reproduction Request
Reply-To: ip@aamc.org

Thank you for your permission request to use the Association of American Medical Colleges (AAMC) copyrighted material. This email provides two actions for you to help us process your request.

1. Below are the most common responses to permission requests. Please review carefully. If your request is addressed below, AAMC will not provide a further response.

Most Common Responses to Permission Requests:

AAMC provides a general permission grant in the AAMC Website Terms and Conditions, please review to determine whether permission has already been granted to you. Topics include scholarly articles, presentations, textbooks, dissertations, and course syllabi.

Many AAMC works include a copyright notice and suggested citation within the work. Please review to determine whether permission has already been granted to you. If the work does not include a suggested citation, AAMC does not have a style preference for proper attribution and citation.

Permission requests for Academic Medicine articles are managed directly through the publisher (and not AAMC): https://journals.lww.com/academicmedicine/_layouts/1033/oaks.journals/rightsandpermissions.aspx.

Permission requests for AAMC MedEdPORTAL material are managed at the resource level because it is an open-access journal and includes a Creative Commons License. If you require further permission, the resource author is the rightsholder.

Permission to link to AAMC material on its website is not required. AAMC generally denies requests to download and repost AAMC material in whole.

The AAMC may only grant permission to its own works. If you are seeking permission to third-party content, you must seek permission from content owner third-party.

2. If your request is not addressed above, respond to this email stating you have reviewed the most common responses and your request requires further assistance. We will evaluate your request and respond within 3-5 business days.

Legal Form Reproductions

Q. First Name:
R. ming

Q. Last Name:
R. lu

Q. Job TItle
R. MD

Q. Email:
R. minglumd@gmail.com

Q. Institution
R. Christus Trinity Clinic Eye Center

AAMC Content Information
Q. Title
R. Stimulation of insulin secretion and insulin gene expression by gastric inhibitory polypeptide

Q. Specific item
R. Figure

Q. Web Address
R.

Intended Use
Q. Describe intended use
R. Use the published figure for a review manuscript

Q. Title of Work
R. Angiogenesis and Inflammation in the Retinopathy Risk of Insulin and Semaglutide

Q. Compiler/Author
R. Ming Lu

Q. Will this work be used for a commercial purpose?
R. No

Q. Publisher(if applicable)
R. Springer Nature

Q. Edition number(if applicable)
R. in review

Q. Publication Date
R. 01/31/2026

Q. Form of reproduction
R. reprint a figure

Q. Intended Audience
R. International Journal of Retina and Vitreous

Q. tfa_noOverWriteFields
R.

Sincerely,
AAMC Copyright Permissions Team
ip@aamc.org

Emails sent to the AAMC are not secure during transit and could be intercepted by others. Do not send sensitive personal information, such as social security numbers (SSNs), health, or credit card information, in response to this email.

ELSEVIER LICENSE
TERMS AND CONDITIONS

Jan 16, 2026

This Agreement between Ming Lu ("You") and Elsevier ("Elsevier") consists of your license details and the terms and conditions provided by Elsevier and Copyright Clearance Center.

License Number	6190640244899
License date	Jan 16, 2026
Licensed Content Publisher	Elsevier
Licensed Content Publication	The Lancet
Licensed Content Title	Intensive blood-glucose control with sulphonylureas or insulin compared with conventional treatment and risk of complications in patients with type 2 diabetes (UKPDS 33)
Licensed Content Date	Sep 12, 1998
Licensed Content Volume	352
Licensed Content Issue	9131

Licensed Content Pages	17
Start Page	837
End Page	853
Type of Use	reuse in a journal/magazine
Requestor type	academic/educational institute
Portion	figures/tables/illustrations
Number of figures/tables/illustrations	1
Format	electronic
Are you the author of this Elsevier article?	No
Will you be translating?	No
Title of new article	Angiogenesis and Inflammation in the Retinopathy Risk of Insulin and Semaglutide
Lead author	ming lu
Title of targeted journal	International Journal of Retina and Vitreous
Publisher	Springer Nature

Publisher imprint	BMC
Expected publication date	Jan 2026
Portions	Figure 7
The Requesting Person / Organization to Appear on the License	Ming Lu
Requestor Location	Dr. Ming Lu<br>1327 Troup Highway<br>Eye Center<br><br>Tyler, TX 75701<br>United States
Order reference number	7
Publisher Tax ID	98-0397604
Total	0.00 USD

Terms and Conditions

INTRODUCTION

1. The publisher for this copyrighted material is Elsevier. By clicking "accept" in connection with completing this licensing transaction, you agree that the following terms and conditions apply to this transaction (along with the Billing and Payment terms and conditions established by Copyright Clearance Center, Inc. ("CCC"), at the time that you opened your RightsLink account and that are available at any time at

https://myaccount.copyright.com).

GENERAL TERMS

2. Elsevier hereby grants you permission to reproduce the aforementioned material subject to the terms and conditions indicated.

3. Acknowledgement: If any part of the material to be used (for example, figures) has appeared in our publication with credit or acknowledgement to another source, permission must also be sought from that source. If such permission is not obtained then that material may not be included in your publication/copies. Suitable acknowledgement to the source must be made, either as a footnote or in a reference list at the end of your publication, as follows:

"Reprinted from Publication title, Vol /edition number, Author(s), Title of article / title of chapter, Pages No., Copyright (Year), with permission from Elsevier [OR APPLICABLE SOCIETY COPYRIGHT OWNER]." Also Lancet special credit - "Reprinted from The Lancet, Vol. number, Author(s), Title of article, Pages No., Copyright (Year), with permission from Elsevier."

4. Reproduction of this material is confined to the purpose and/or media for which permission is hereby given. The material may not be reproduced or used in any other way, including use in combination with an artificial intelligence tool (including to train an algorithm, test, process, analyse, generate output and/or develop any form of artificial intelligence tool), or to create any derivative work and/or service (including resulting from the use of artificial intelligence tools).

5. Altering/Modifying Material: Not Permitted. However figures and illustrations may be altered/adapted minimally to serve your work. Any other abbreviations, additions, deletions and/or any other alterations shall be made only with prior written authorization of Elsevier Ltd. (Please contact Elsevier's permissions helpdesk here). No modifications can be made to any Lancet figures/tables and they must be reproduced in full.

6. If the permission fee for the requested use of our material is waived in this instance, please be advised that your future requests for Elsevier materials may attract a fee.

7. Reservation of Rights: Publisher reserves all rights not specifically granted in the combination of (i) the license details provided by you and accepted in the course of this licensing transaction, (ii) these terms and conditions and (iii) CCC's Billing and Payment terms and conditions.

8. License Contingent Upon Payment: While you may exercise the rights licensed

immediately upon issuance of the license at the end of the licensing process for the transaction, provided that you have disclosed complete and accurate details of your proposed use, no license is finally effective unless and until full payment is received from you (either by publisher or by CCC) as provided in CCC's Billing and Payment terms and conditions. If full payment is not received on a timely basis, then any license preliminarily granted shall be deemed automatically revoked and shall be void as if never granted. Further, in the event that you breach any of these terms and conditions or any of CCC's Billing and Payment terms and conditions, the license is automatically revoked and shall be void as if never granted. Use of materials as described in a revoked license, as well as any use of the materials beyond the scope of an unrevoked license, may constitute copyright infringement and publisher reserves the right to take any and all action to protect its copyright in the materials.

9. Warranties: Publisher makes no representations or warranties with respect to the licensed material.

10. Indemnity: You hereby indemnify and agree to hold harmless publisher and CCC, and their respective officers, directors, employees and agents, from and against any and all claims arising out of your use of the licensed material other than as specifically authorized pursuant to this license.

11. No Transfer of License: This license is personal to you and may not be sublicensed, assigned, or transferred by you to any other person without publisher's written permission.

12. No Amendment Except in Writing: This license may not be amended except in a writing signed by both parties (or, in the case of publisher, by CCC on publisher's behalf).

13. Objection to Contrary Terms: Publisher hereby objects to any terms contained in any purchase order, acknowledgment, check endorsement or other writing prepared by you, which terms are inconsistent with these terms and conditions or CCC's Billing and Payment terms and conditions. These terms and conditions, together with CCC's Billing and Payment terms and conditions (which are incorporated herein), comprise the entire agreement between you and publisher (and CCC) concerning this licensing transaction. In the event of any conflict between your obligations established by these terms and conditions and those established by CCC's Billing and Payment terms and conditions, these terms and conditions shall control.

14. Revocation: Elsevier or Copyright Clearance Center may deny the permissions described in this License at their sole discretion, for any reason or no reason, with a full refund payable to you. Notice of such denial will be made using the contact information provided by you. Failure to receive such notice will not alter or invalidate the denial. In no event will Elsevier or Copyright Clearance Center be responsible or liable for any

costs, expenses or damage incurred by you as a result of a denial of your permission request, other than a refund of the amount(s) paid by you to Elsevier and/or Copyright Clearance Center for denied permissions.

LIMITED LICENSE

The following terms and conditions apply only to specific license types:

15. **Translation**: This permission is granted for non-exclusive world **English** rights only unless your license was granted for translation rights. If you licensed translation rights you may only translate this content into the languages you requested. A professional translator must perform all translations and reproduce the content word for word preserving the integrity of the article.

16. **Posting licensed content on any Website**: The following terms and conditions apply as follows: Licensing material from an Elsevier journal: All content posted to the web site must maintain the copyright information line on the bottom of each image; A hyper-text must be included to the Homepage of the journal from which you are licensing at http://www.sciencedirect.com/science/journal/xxxxx or the Elsevier homepage for books at http://www.elsevier.com; Central Storage: This license does not include permission for a scanned version of the material to be stored in a central repository such as that provided by Heron/XanEdu.

Licensing material from an Elsevier book: A hyper-text link must be included to the Elsevier homepage at http://www.elsevier.com . All content posted to the web site must maintain the copyright information line on the bottom of each image.

Posting licensed content on Electronic reserve: In addition to the above the following clauses are applicable: The web site must be password-protected and made available only to bona fide students registered on a relevant course. This permission is granted for 1 year only. You may obtain a new license for future website posting.

17. **For journal authors:** the following clauses are applicable in addition to the above:

Preprints:

A preprint is an author's own write-up of research results and analysis, it has not been peer-reviewed, nor has it had any other value added to it by a publisher (such as formatting, copyright, technical enhancement etc.).

Authors can share their preprints anywhere at any time. Preprints should not be added to

or enhanced in any way in order to appear more like, or to substitute for, the final versions of articles however authors can update their preprints on arXiv or RePEc with their Accepted Author Manuscript (see below).

If accepted for publication, we encourage authors to link from the preprint to their formal publication via its DOI. Millions of researchers have access to the formal publications on ScienceDirect, and so links will help users to find, access, cite and use the best available version. Please note that Cell Press, The Lancet and some society-owned have different preprint policies. Information on these policies is available on the journal homepage.

Accepted Author Manuscripts: An accepted author manuscript is the manuscript of an article that has been accepted for publication and which typically includes author-incorporated changes suggested during submission, peer review and editor-author communications.

Authors can share their accepted author manuscript:

- immediately
  - via their non-commercial person homepage or blog
  - by updating a preprint in arXiv or RePEc with the accepted manuscript
  - via their research institute or institutional repository for internal institutional uses or as part of an invitation-only research collaboration work-group
  - directly by providing copies to their students or to research collaborators for their personal use
  - for private scholarly sharing as part of an invitation-only work group on commercial sites with which Elsevier has an agreement
- After the embargo period
  - via non-commercial hosting platforms such as their institutional repository
  - via commercial sites with which Elsevier has an agreement

In all cases accepted manuscripts should:

- link to the formal publication via its DOI
- bear a CC-BY-NC-ND license - this is easy to do
- if aggregated with other manuscripts, for example in a repository or other site, be shared in alignment with our hosting policy not be added to or enhanced in any way to appear more like, or to substitute for, the published journal article.

Published journal article (JPA): A published journal article (PJA) is the definitive final record of published research that appears or will appear in the journal and embodies all value-adding publishing activities including peer review co-ordination, copy-editing, formatting, (if relevant) pagination and online enrichment.

Policies for sharing publishing journal articles differ for subscription and gold open access articles:

Subscription Articles: If you are an author, please share a link to your article rather than the full-text. Millions of researchers have access to the formal publications on ScienceDirect, and so links will help your users to find, access, cite, and use the best available version.

Theses and dissertations which contain embedded PJAs as part of the formal submission can be posted publicly by the awarding institution with DOI links back to the formal publications on ScienceDirect.

If you are affiliated with a library that subscribes to ScienceDirect you have additional private sharing rights for others' research accessed under that agreement. This includes use for classroom teaching and internal training at the institution (including use in course packs and courseware programs), and inclusion of the article for grant funding purposes.

Gold Open Access Articles: May be shared according to the author-selected end-user license and should contain a CrossMark logo, the end user license, and a DOI link to the formal publication on ScienceDirect.

Please refer to Elsevier's posting policy for further information.

18. **For book authors** the following clauses are applicable in addition to the above: Authors are permitted to place a brief summary of their work online only. You are not allowed to download and post the published electronic version of your chapter, nor may you scan the printed edition to create an electronic version. **Posting to a repository:** Authors are permitted to post a summary of their chapter only in their institution's repository.

19. **Thesis/Dissertation**: If your license is for use in a thesis/dissertation your thesis may be submitted to your institution in either print or electronic form. Should your thesis be published commercially, please reapply for permission. These requirements include permission for the Library and Archives of Canada to supply single copies, on demand, of the complete thesis and include permission for Proquest/UMI to supply single copies, on demand, of the complete thesis. Should your thesis be published commercially, please reapply for permission. Theses and dissertations which contain embedded PJAs as part of the formal submission can be posted publicly by the awarding institution with DOI links back to the formal publications on ScienceDirect.

Elsevier Open Access Terms and Conditions

You can publish open access with Elsevier in hundreds of open access journals or in nearly 2000 established subscription journals that support open access publishing. Permitted third party re-use of these open access articles is defined by the author's choice of Creative Commons user license. See our open access license policy for more information.

Terms & Conditions applicable to all Open Access articles published with Elsevier:

Any reuse of the article must not represent the author as endorsing the adaptation of the article nor should the article be modified in such a way as to damage the author's honour or reputation. If any changes have been made, such changes must be clearly indicated.

The author(s) must be appropriately credited and we ask that you include the end user license and a DOI link to the formal publication on ScienceDirect.

If any part of the material to be used (for example, figures) has appeared in our publication with credit or acknowledgement to another source it is the responsibility of the user to ensure their reuse complies with the terms and conditions determined by the rights holder.

Additional Terms & Conditions applicable to each Creative Commons user license:

CC BY: The CC-BY license allows users to copy, to create extracts, abstracts and new works from the Article, to alter and revise the Article and to make commercial use of the Article (including reuse and/or resale of the Article by commercial entities), provided the user gives appropriate credit (with a link to the formal publication through the relevant DOI), provides a link to the license, indicates if changes were made and the licensor is not represented as endorsing the use made of the work. The full details of the license are available at http://creativecommons.org/licenses/by/4.0.

CC BY NC SA: The CC BY-NC-SA license allows users to copy, to create extracts, abstracts and new works from the Article, to alter and revise the Article, provided this is not done for commercial purposes, and that the user gives appropriate credit (with a link to the formal publication through the relevant DOI), provides a link to the license, indicates if changes were made and the licensor is not represented as endorsing the use made of the work. Further, any new works must be made available on the same conditions. The full details of the license are available at http://creativecommons.org/licenses/by-nc-sa/4.0.

CC BY NC ND: The CC BY-NC-ND license allows users to copy and distribute the

Article, provided this is not done for commercial purposes and further does not permit distribution of the Article if it is changed or edited in any way, and provided the user gives appropriate credit (with a link to the formal publication through the relevant DOI), provides a link to the license, and that the licensor is not represented as endorsing the use made of the work. The full details of the license are available at http://creativecommons.org/licenses/by-nc-nd/4.0. Any commercial reuse of Open Access articles published with a CC BY NC SA or CC BY NC ND license requires permission from Elsevier and will be subject to a fee.

Commercial reuse includes:

- Associating advertising with the full text of the Article
- Charging fees for document delivery or access
- Article aggregation
- Systematic distribution via e-mail lists or share buttons

Posting or linking by commercial companies for use by customers of those companies.

20. **Other Conditions**:

v1.11

Questions? customercare@copyright.com.
